# Carbonylation of Ethene Catalysed by Pd(II)-Phosphine Complexes

**DOI:** 10.3390/molecules190915116

**Published:** 2014-09-22

**Authors:** Gianni Cavinato, Luigi Toniolo

**Affiliations:** 1 Department of Chemical Sciences, University of Padua, Padua 35131, Italy; E-Mail: gianni.cavinato@unipd.it; 2 Department of Molecular Sciences and Nanosystems, Ca’ Foscari University of Venice, Venice 30123, Italy

**Keywords:** carbonylation, ethene, palladium-phosphine, catalysts

## Abstract

This review deals with olefin carbonylation catalysed by Pd(II)-phosphine complexes in protic solvents. In particular, the results obtained in the carbonylation with ethene are reviewed. After a short description of the basic concepts relevant to this catalysis, the review treats in greater details the influence of the bite angle, skeletal rigidity, electronic and steric bulk properties of the ligand on the formation of the products, which range from high molecular weight perfectly alternating polyketones to methyl propanoate. It is shown that the steric bulk plays a major role in directing the selectivity. Particular emphasis is given to the factors governing the very active and selective catalysis to methyl propanoate, including the mechanism of the catalytic cycles with diphosphine- and monophosphine-catalysts. A brief note on the synthesis of methyl propanoate using a “Lucite” type catalyst in ionic liquids is also illustrated. A chapter is dedicated to the carbonylation of olefins in aqueous reaction media. The nonalternating CO-ethene copolymerization is also treated.

## 1. Introduction

Palladium catalysed olefin carbonylation has been of industrial interest for about five decades, as documented by several patents. An earlier publication reported that Pd(II)-monophosphine complexes of the type [PdCl_2_(PPh_3_)_2_], in combination with HCl, were active though under rather severe pressure conditions (300–700 bar). The carbonylation of ethene in EtOH gave ethyl propanoate. No mention of other carbonylated products was reported [1]. Subsequently, it was found that the reaction could be carried out at a significantly lower pressure. This fact prompted several research groups also from academia to get involved. The carbonylation was extended to other olefinic substrates using Pd(II) catalysts with a large variety of ligands. The subject was reviewed [2,3,4,5]. In the early 1980s, Sen * et al.* reported that cationic complexes of the general formula [Pd(PPh_3_)_n_(CH_3_CN)_4-n_](BF_4_)_2_ (n = 1–3) were active even at room conditions. In CH_2_Cl_2_ or CHCl_3_ as solvents, a high molecular weight alternating CO-ethene polyketone (PK) was obtained, whereas in MeOH the main product was methyl propanoate (MP) which formed together with alternating cooligomers of the general formula H(CH_2_CH_2_CO)_n_OMe. The corresponding neutral complex [PdCl_2_(PPh_3_)_2_] was ineffective, indicating that the vacancy of coordination sites, created by the dissociation of the weakly coordinating ligands, played a key role [6,7]. In the same period Drent *et al.* of the Shell company patented the discovery that substitution of the monophosphine ligand with a diphosphine one, in particular 1,3-*bis*(phosphino)propanes, led to highly active catalysts for the copolymerization reaction and that the terpolymerization with propene gave a material of with a lower melting point with improved stability and rheology. These facts, the ready availability of the monomers at a relatively low cost and the interesting properties of PKs that make them suitable for many applications, made the production of these materials attractive. Shortly after disclosing the basic aspects of this chemistry [8], many academic research groups also became involved in this fascinating chemistry. The results have been widely reviewed [9,10,11,12,13,14,15,16,17,18], as well as stereochemical copolymerization, synthesis of chiral, optically active copolymers, and theoretical studies [19,20,21]. Copolymerization has been extended to styrene and its derivatives, which requires the use of Pd(II) catalysts with chelating N-N ligands [17,18,19,20,22,23], and to functionalized olefins [24].

The carbonylation of ethene is of interest not only for making PKs, but also for the production of methyl propanoate (MP), an intermediate for the environmentally friendly synthesis of methyl methacrylate, a high-value monomer for the production of signs, coatings, adhesives, fibres, paints and so-called organic glasses.

Although the carbonylation reactions may be carried out in aprotic solvents [25,26,27,28,29,30,31], most of the studies have been carried out in MeOH. This review will mainly deal with the mechanistic aspects of ethene carbonylation catalysed by Pd(II)-P complexes (P = mono- or di-phosphine), starting from the chemistry relevant to in the formation of a PK in MeOH because the development of this chemistry led to the discovery of highly efficient catalytic systems for the synthesis of MP (*cfr.* Section 3.1., Section 4. and Section 5.1.). Investigations in other protic solvents will also be presented. With the purpose of facilitating the understanding of more recent findings, the basic aspects previously reviewed will first be concisely considered.

## 2. Basic Concepts of CO-Ethene Copolymerization in MeOH

### 2.1. General Aspects

**Scheme 1:**
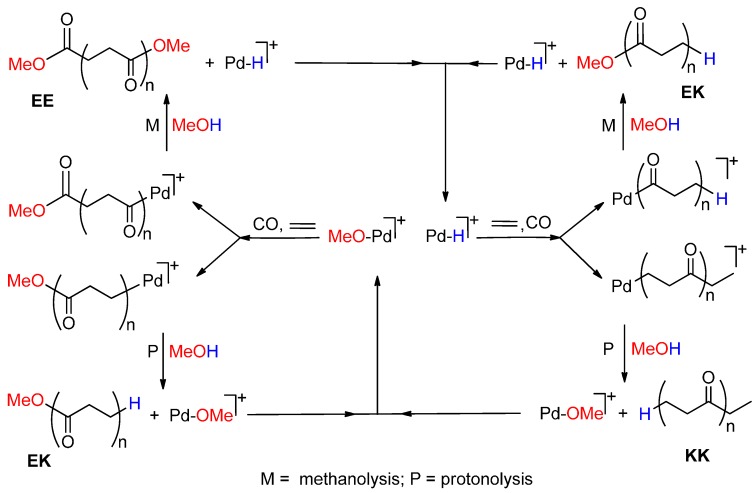
Catalytic cycles for the CO-ethene copolymerization in MeOH.

The general aspects outlined in early publications are still valid, though, luckily, with some important exceptions. What became soon of general consensus was:

(i) Monophosphine catalysts lead to MP and eventually to low molecular weight co-oligomers, whereas diphosphine ones give PKs. Diphosphines, having the two P atoms linked by a C_3_ chain, give PKs with higher productivities and molecular weights than the others [8,10]. The striking difference between monophosphine- and diphosphine-catalysts was explained as the result that the diphosphine is always *cis*-chelated, so that the other two coordination sites of the *d*^8^-square planar palladium centre, one occupied by the growing polymer chain and the other by the monomer, are also always *cis* to each other, which is ideal in favouring the chain growth through migratory insertions of the monomers. Differently, Pd(II) coordinated by a monophosphine can assume both *cis* and *trans* geometries, so that chain growth is relatively slow compared to chain termination [8,10].
(ii) Only “cationic” catalysts in which the charge of the central Pd(II) is balanced by weakly coordinating anions are highly active [6,8,9,10].
(iii) Alternating PKs are formed because the consecutive insertion of two molecules of CO is not allowed for thermodynamic reasons and two consecutive insertions of ethene do not occur for kinetic reasons [9,10,32,33].
(iv) The formation of the PK occurs through both Pd-H^+^ and Pd-OMe^+^ initiators, as unambiguously proven by the end-group analysis of the PK. The initiators are re-generated in the chain-transfer process, which occurs by MeOH protonolysis and methanolysis [8,10] (Scheme 1).

Thus the PK consists of a mixture of three PKs, differing only in the keto (CH_3_CH_2_CO‑) or ester (‑COOCH_3_) end-groups, indicated with **K** and **E**, respectively. Hereafter, the three polymers are named **EK**, **KK** and **EE**. The cycles leading to **EE** and **KK** PKs are interconnected [8,10]. The crossover between them has been demonstrated [34,35] (*cfr*. Section 2.4.). Quite interestingly, when using the monophosphine-catalyst [Pd(PPh_3_)_n_(CH_3_CN)_4-n_](BF_4_)_2_ (n = 1–3), the low molecular weight PK is formed through a cycle in which the termination occurs exclusively via methanolysis with re-generation of the Pd-H^+^ initiator, so that only one type of copolymer is formed, *i.e.*, **EK** [7].

### 2.2. Alternating Chain Growing

**Scheme 2:**
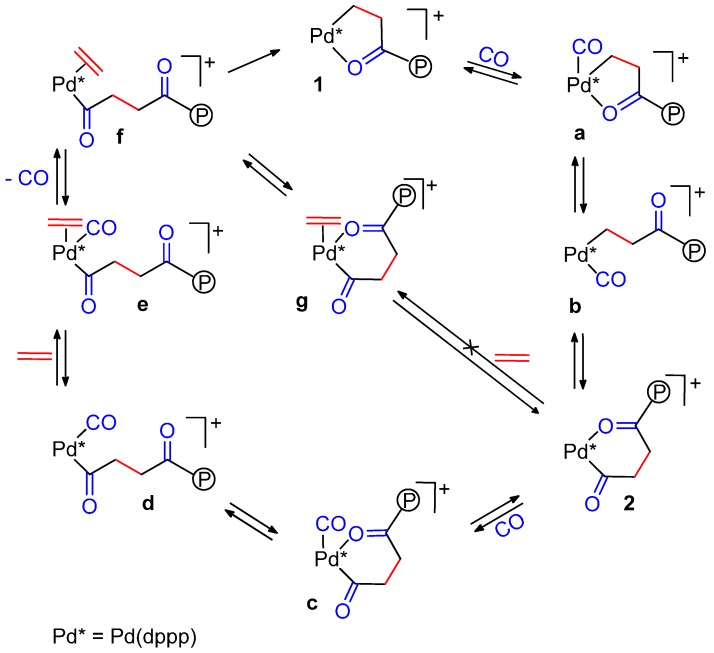
Alternating chain growing process for the CO-ethene copolymerization: detected intermediates **1** and **2**; proposed ones **a**–**f**.

By the use of polarization modulation reflection absorption infrared spectroscopy, Drent *et al.* monitored in the microcrystalline state the single insertion steps starting from [Pd(CH_3_)(OTf)(dppp)]. The presence of β- and γ-chelates **1** and **2** was detected (Scheme 2).

Having a higher binding affinity, CO inserts into a five-membered β-chelate to give a six-membered γ-chelate. Since a five-membered ring is more stable than a six-membered one, further propagation occurs with the insertion of ethene with formation of the next five-membered ring. This explains the perfect alternation [36]. It was also found that substitution of the chelating keto group by ethene is CO-assisted and occurs in two steps, **2** → **c** → **d** and **d** → **e** → **f**, and that the substitution of the chelating keto group by CO is more facile than by ethene for steric reasons. Another finding was that the total abundance of **1** and **2** and their ratio remain constant, so that both are resting states [36]. From thermodynamic and kinetic data, Brookhart *et al.* calculated that the insertion of ethene into a Pd-alkyl bond (double ethene insertion) could occur every *ca.* 10^5^ CO insertions into the same bond, which accounts for the strictly alternating chain growth [37].

**Scheme 3:**
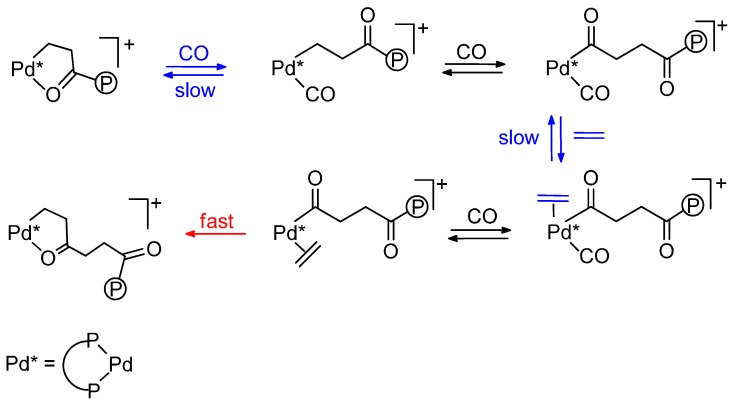
Slow steps in the CO-ethene copolymerization.

Such chelates have also been detected in model reactions through variable temperature multinuclear NMR spectroscopy, thus the above mechanism is basically operating also in solution [13,34,35,37,38,39]. From kinetic and thermodynamic data, it was proposed that the slow step is not a single step, but rather it seems to be the opening of a β-chelate by CO, followed by ethene displacement to give the next β-chelate [13] (Scheme 3).

Theoretical studies are in line with this suggestion, as it was found that, although ethene insertion into Pd-acyl bonds is facile, the relatively low affinity of the metal centre for coordination of the electron donating ethene ligand compared to CO makes the overall process of ethene insertion slow [40], as initially assumed [10].

### 2.3. Termination-Initiation

**Figure 1:**
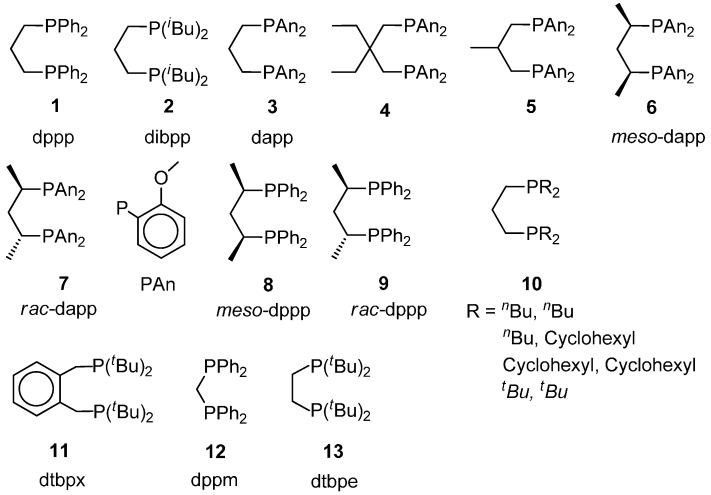
P-P ligands for the carbonylation of ethene.

**Scheme 4:**
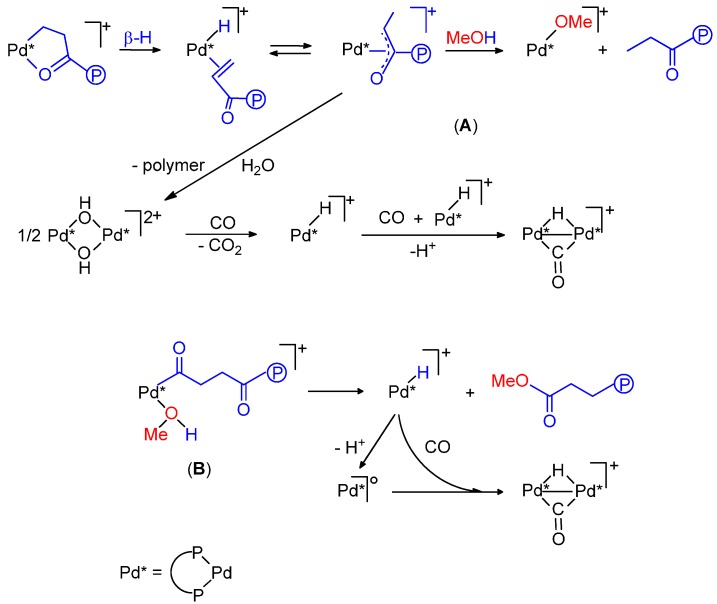
Chain-transfer mechanisms for the CO-ethene copolymerization: protonolysis with MeOH (**A**); methanolysis (**B**).

**Figure 2:**
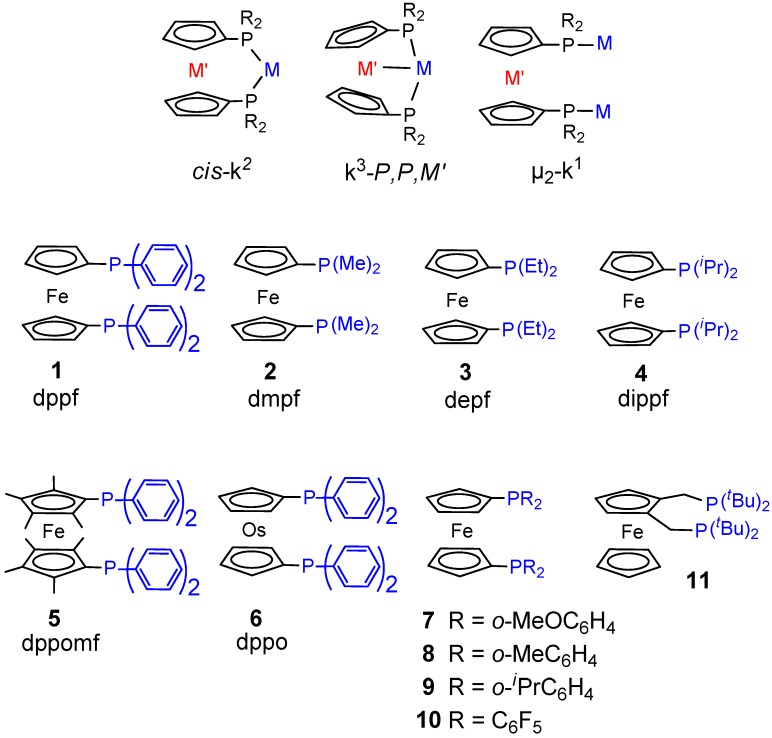
Metallocene-coordination modes and -ligands.

Chain transfer occurs via protonolysis with MeOH or methanolysis. Model reactions with CH_3_OD have shown that protonolysis involves a preequilibrium of the β-chelate with its enolate isomer through a slow β-H elimination/fast hydride re-insertion with formation of an enolate, which is protonated to give an enol, which rearranges to the keto-end group (Scheme 4A) [38]. This mechanism is operative also in the hydroacylation of ethene to light products like diethylketone (DEK) [41,42]. Methanolysis studies of complexes of the type [Pd(COMe)(P-P)]^+^ led to the conclusion that (i) Coordination of MeOH *cis* to the acyl ligand is required and (ii) The rate of methanolysis increases with increasing steric bulk [43]. The fact that coordination of MeOH to palladium is an essential prerequisite to methanolysis has been evidenced also by studying the effect of solvent, counter-anion and occupancy of the fourth site on [Pd(dibpp)(COCH_3_)L]^n+^ (dibpp = ligand 2 in Figure 1; n = 0 or 1; L = counter-anion or CH_3_CN, CO) [44]. However, there are cases in which methanolysis may occur intermolecularly. As a matter of fact, the complex [Pd(dppomf)(COCH_3_)]^+^ (dppomf = ligand **5** in Figure 2) undergoes immediate methanolysis at room temperature and even though (i) The dppomf acts as a tridentate η^3^-P, P, Fe ligand; with *trans* P atoms and (ii) Ethene does not insert, which is indicative that a coordination site is not easily available. Thus, in this case, methanolysis may occur intermolecularly [45]. Intermolecular methanolysis may also occur in the hydromethoxycarbonylation of ethene catalysed by the osmocene analogue of dppf [46] (see also Section 3.1.). For the nature of these less common ligands see Figure 2.

Ideally, the Pd-OCH_3_^+^ or Pd-H^+^ species that initiate the catalytic cycle are re-generated at the termination step of the chain propagation process. However, the hydride that is formed in the methanolysis step may deprotonate with formation of less active Pd(0) and dimer species (Scheme 4B) [10,11,43]. Efficient catalytic activity is achieved by using the precursor in combination with an acid, to stabilize the hydride as well as to convert less active μ-OH dimers (generated by adventitious water) to active mononuclear species [47] or an oxidant for the re-generation of active Pd(II) species from Pd(I) or Pd(0) species that eventually form during the course of catalysis [10,11,13].

### 2.4. Shift from the Hydride Mechanism to the Methoxy One and Vice Versa

The Pd-H^+^ generated in the methanolysis step may be transformed into Pd-OCH_3_^+^, when a quinone is used. *Vice versa*, the Pd-OCH_3_^+^ generated in the protonolysis step may undergo β-H elimination with formation of Pd-H^+^, as it occurs for the hydroacylation of ethene to DEK [42]. Even when these transformations do not occur, the shift from the Pd-H route to the Pd-OCH_3_ route occurs because chain terminations yield, in addition to the PK, both initiators.

**Scheme 5:**
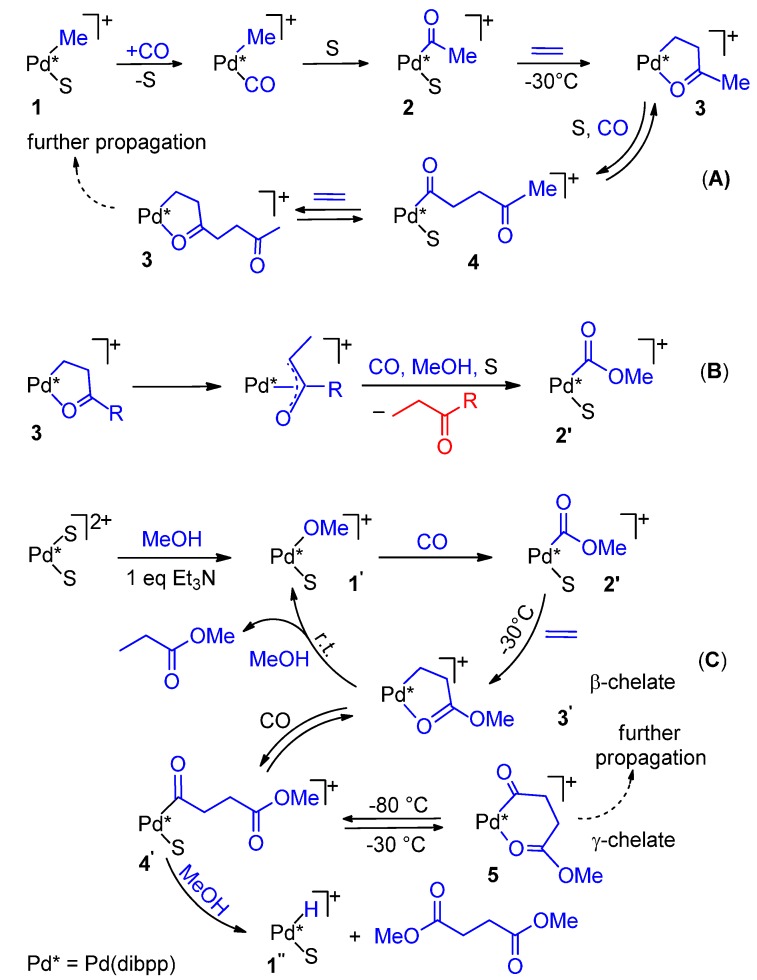
The Pd-H (Pd-Me) cycle (**A**); shift from the Pd-Me (Pd-H) route to the Pd-OMe one (**B**); the Pd-OMe cycle (**C**).

Direct shift from the hydride mechanism to the carbomethoxy one has been demonstrated by Iggo *et al.* by multinuclear NMR spectroscopy [34,35]. The methyl complex [Pd(CH_3_)(CH_3_CN)(dibpp)](TfO) was used as a Pd-H initiator (Scheme 5A), because attempts to prepare a corresponding Pd-hydride were unsuccessful.

In Scheme 5B the shift from the hydride mechanism to the other mechanism is shown to occur via an enolate intermediate. If in the cycle initiated by a Pd-OCH_3_ species, termination occurs via methanolysis of a γ-chelate (or its open isomer), then the cycle is shifted to the other (Scheme 5C, steps **1'** **→** **2'→** **3'→** **4'→** **1''**).

In the hydride pathway, it has been found that protonolysis of the third-generation β-chelate at r.t. is significantly slower than that of a second generation β-chelate. This fact would imply that this termination becomes even slower as the chain growing goes on, thus favouring the formation of higher products. It was also found that protonolysis of the β-ester chelate **5** is very slow, which also favours the propagation process.

## 3. Influence of the Ligand on the Product Formation

The carbonylation products of ethene range from high molecular weight PKs to MP, which can be considered as the first member of the CO-ethene copolymerization process. Since the discovery of highly efficient catalysts for the production of PKs predates that for making MP, this chapter deals first with the main factors that influence the copolymerization process. For a given catalyst, productivity and molecular weight of the obtained PK depend on the operative conditions, *i.e.*, temperature, pressure, monomer ratio, reaction time, solvent, counteranion, as well as the presence of promoters [8,10,47,48,49,50,51,52,53], but it is the nature of the ligand that can exert a dramatic effect.

### CO-Ethene Chain Propagation

It was found by Drent *et al.* that among the ligands of the series Ph_2_P(CH_2_)_n_PPh_2_ (n = 1–6), the one with n = 3 is the most effective in terms of both productivity and molecular weight. It was proposed that the energy barrier for the insertion reactions is lower with a diphosphine having a natural bite angle close to 90°, which is better satisfied with n = 3 [8,10].

According to this conviction, the dppp ligand has been modified with substituents at the phenyl ring and with alkyl substitution in the bridging chain (ligands **3**–**7** in Figure 1). *o*- and *p-*methoxy substitution at the phenyl rings enhances both the productivity as well as the molecular weight. The close performance of the so-modified dppp catalysts suggests that the main effect is of an electronic nature. It was later found that, even though no direct evidence for the coordination of the oxygen atoms of the methoxy groups with palladium have been observed, NMR spectroscopy experiments have shown that the aryl rings adopt a conformation which favours the MeO-Pd interactions, thus facilitating the opening of a β-chelate ring by CO compared to the dppp-based catalyst [54].

The effect of steric hindrance caused by stereochemical rigidity is illustrated by methyl substitution at the bridging chain of dppp, particularly at the 1- and 3-carbon atoms in *meso*-position (ligand **8**) [55]. It was suggested that because of the higher rigidity in the bridging chain the *meso* ligand assumes a conformation of higher steric hindrance, which destabilizes the β-chelate ring, thus favouring the chain-propagation process. The positive *meso*-effect has also been found in the methyl substituted dapp ligand **6** [56].

**Figure 3:**
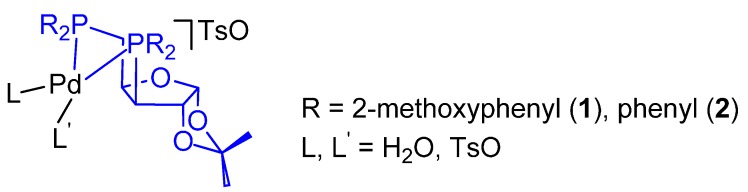
Palladium complexes with *o*-MeO-xylophos and xylophos.

The catalysts reported in Figure 3 have been used for the CO-ethene and CO-propene copolymerization as well as for the CO-ethene-propene terpolymerization in MeOH. The catalytic activity and the molecular weight of the PKs have been compared to those of the PKs obtained with analogous catalysts with ligands **1** and **3** of Figure 1 [57]. It has been found that: (i) the presence of *o*-MeO substituents on the P-phenyl rings and of a high conformational rigidity of the ligand exerts a negative influence on the activity differently from the catalyst with ligand **3**; (ii) in the CO-ethene copolymerization, the molecular weight decreases by decreasing the basicity of the ligand (as the termination rate by either methanolysis or protonolysis increases with the electrophilicity of the metal centre) and increases by decreasing the skeletal rigidity as the propagation chain is favoured; (iii) neither the substitution of phenyl with 2-methoxyphenyl nor the introduction of an α-d-xylofuranosyl carbon backbone increases the low regioselectivity of propene insertion as compared to the dppp-based catalyst [58,59]; (iv) With ligand **1** the productivity is comparable to that achieved with the ligand **3**-based industrial catalyst at a higher pressure (72 bar) [60], but it promotes a higher propene incorporation.

As already mentioned, after Drent rationalized the results on the copolymerization process, it was generally accepted for several years that monophsphine catalysts lead to MP, whereas *cis*-chelating ones promote the multiple monomer insertions leading to copolymers. Later, Drent himself found that substitution of the phenyl rings with alkyl groups of increasing steric bulk (ligand **10**) led to the formation of lighter products to the point that when R = *^t^*Bu, MP was produced with 97.4% selectivity at 120 °C, 40 bar, CO:ethene = 2:1, TOF = 25,000 h^−1^ [42]. Even more selective was the catalyst based on the dtbpx ligand **11** under milder conditions (selectivity 99.9%, TOF = 12,000 h^−1^, at 80 °C, 10 bar, CO:ethene = 1:1) [61].

The idea that only monophosphines are more suitable to yield MP was hard to comprehend. As a matter of fact, at first, it was suggested that the dtbpx ligand opened one coordination arm and acted as a monophosphine. However, it has been proven by multinuclear NMR spectroscopy that the ligand remains *cis*-coordinated throughout the entire catalytic cycle [62,63,64,65] (see Section 5.1. below).

The dppm-derived catalysts are also illustrative of the effect of steric hindrance. The dppm ligand **12** bridges two Pd atoms with mutually *trans* P atoms on each metal [66], which may explain their low activity in the copolymerization reaction. By introducing bulky alkyl groups in the *ortho*-position of the phenyl rings, the ligand acts preferentially as a *cis*-coordinating bidentate on one Pd centre and gives rise to a very active catalyst for the production of PKs [67].

The case of the [PdCl_2_(PPh_3_)_2_]/PPh_3_/HCl system is also noteworthy. In MeOH, it was expected to give MP at a low rate, because the metal is coordinated by a monophosphine and a strongly coordinating chloride anion. Instead, it has been found that at 100 °C, 60 bar total pressure, in the presence of an excess of HCl (initial HCl:Pd = 80:1), a PK is formed at a comparable rate of MP (TOF *ca*. 150 as (moles of ethene absorbed)(mol·Pd·h)^−1^, whereas when ethene:CO = 40:20, the main product is a PK of moderate M_n_ (2000–2500 g·mol^−1^, TOF = 300 h^−1^), the formation of MP being almost suppressed. It was suggested that the acid, in addition to stabilizing the Pd-H initiator, could also destabilize the β- and γ-chelates through protonation of the oxygen atoms coordinating the metal centre, thus easing the chain growing process [68].

**Figure 4:**
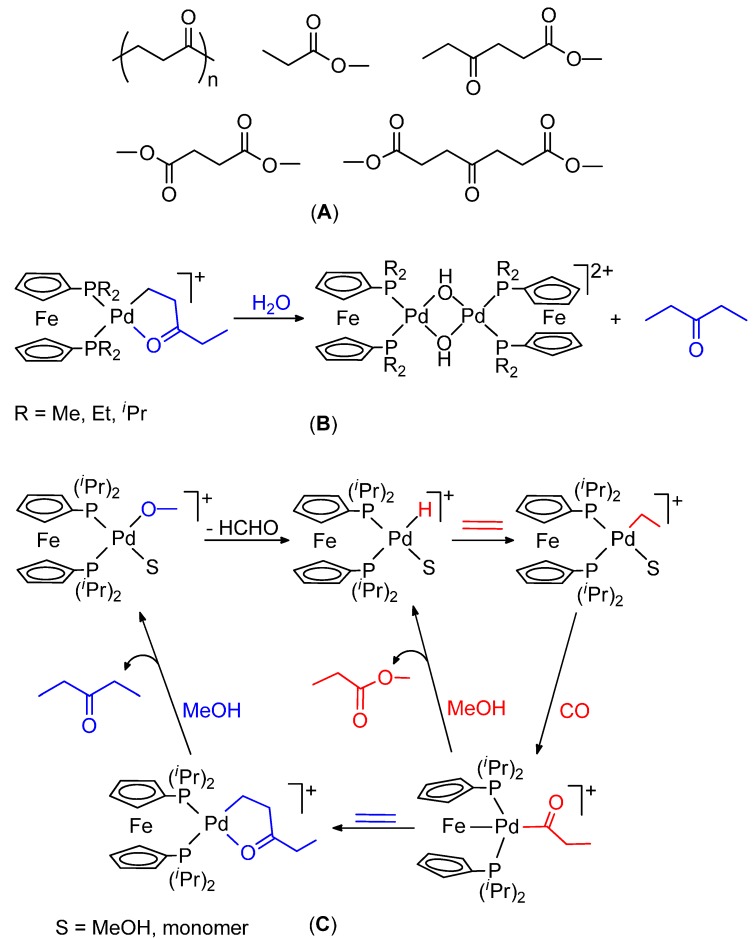
Light products of the ethene carbonylation catalysed by a dppf-based system (**A**); hydrolysis of a β-chelate (**B**); catalytic cycles for the formation of MP and DEK (**C**).

It is also worth reporting the results on 1,1'-*bis*(diorganylphosphino)metallocene-based catalysts. These less traditional ligands are of much interest because their catalytic activity is very sensitive to their different structural and electronic characteristics, which depends on the substituents on the Cp rings, on the P atoms and on the nature of the metal centre. They assume three bonding modes *cis*-κ^2^-P,P, κ^3^-P,P,Fe and μ_2_-κ^1^-P (Figure 2). The activity of catalysts based on dppf, dmpf, depf, dippf (Figure 2) has been studied [45,69]. These ligands present a basicity in the order: dppf < dmpf < depf < dippf. This is also the order of their increasing steric hindrance. The results may be summarized as follows: (i) the carbonylation of ethene in MeOH with the dppf catalyst yields a wide spectrum of products, from monocarbonylated ones to light PKs (Figure 4A); (ii) the dmpf and depf catalysts produce exclusively high molecular weight PKs and the productivity of the depf is higher than that of the other; (iii) the dppf catalyst is less productive and less stable than the dmpf and depf ones; (iv) the higher productivity and stability of the dmpf and depf catalysts are ascribed to their higher basicity; (v) the dmpf catalyst is highly active in the presence of benzoquinone (BQ) and 4-toluenesulfonic acid (TsOH), whereas the depf catalyst does not require their use; (vi) the molecular weight of the PK obtained with the dmpf catalyst is higher than that obtained with the other; (vii) the dippf catalyst is much less active than all the others and is selective to MP and DEK, which are formed at comparable rates; (viii) the presence of β-chelates has been detected with all the catalysts; (ix) the dmpf and depf catalysts are efficient also for the terpolymerization of CO with ethene and propene in MeOH or H_2_O.

The reaction of the β-chelates with water occurs with formation of [Pd(μ-OH)(P-P)]_2_^2+^ and 2-butanone, with rates in the order dmpf < depf < dippf (Figure 4B). The faster reaction of the depf β-chelate compared with that of the dmpf one is in line with the lower molecular weight of the PK yielded by the depf catalyst. This, and the fact that this catalyst is more productive than the other, indicate that the rate of the chain-growing process is higher with the depf system and that the rate of the termination step increases even more. Moreover, these results suggest that steric factors may play some role: bulkier depf ligand destabilizes the β-chelate to a higher extent, thus making the chain growing process faster (higher productivity), but at the same time also favouring the termination step (lower molecular weight).

Steric factors apparently play a major role in the case of the dippf catalysis, which produces 3-pentanone (DEK) and MP at comparable rates, indicating that catalysis undergoes via a common intermediate involved in the slow step. The formation of DEK occurs exclusively through a Pd-H^+^ initiator, which inserts ethene and then CO to give a Pd-acyl intermediate. At this point, further insertion of the olefin yields a keto β-chelate, which undergoes protonolysis with MeOH to giving DEK and a Pd-OCH_3_^+^ species, which re-generates the Pd-H^+^ through β-H abstraction. This initiator is also formed by methanolysis of the Pd-acyl intermediate, which gives MP.

The common intermediate may be a Pd-ethyl or a Pd-acyl species (Figure 4C). An electronic withdrawing ligand such as the acyl one and the steric hindrance at the P atoms may force the ligand to adopt the κ^3^-P,P,Fe coordination mode. It was suggested that the formation of the dative Fe-Pd bond may be the factor that controls the selective formation of the two products and that the κ^3^-P,P,Fe acyl species is the common intermediate. Since the two products are formed at comparable rates, it was concluded that the slow step of the two catalytic cycles may be the hapticity change of dippf from κ^3^-P,P,Fe to κ^2^-P,P, because both the methanolysis of the acyl intermediate or the insertion of ethene require *cis*-P atoms to occur [69].

The fact that the steric hindrance induces a κ^3^-P,P,Fe bonding mode and plays a key role in directing the catalysis is also supported by the results obtained with the dppomf-based catalyst (ligand **5**, Figure 2), which produces selectively MP [45,70]. In both dippf-Pd and dppomf-Pd systems the crystallographic P-Pd-P bite angle (*ca*. 102°–103°) is significantly larger than in the dppf- (96°) and in the dmpf-analogous (92°).

Another interesting case is that of the catalyst with osmocene (ligand **6**, Figure 2), which also produces selectively MP. NMR studies on [PdX(dppo)]TsO (X = TsO, Me, COMe) are consistent with the presence of Os-Pd interactions. X-ray studies on [Pd(CH_3_CN)(dppo)(TsO)_2_] have shown the presence of a strong Pd-Os bond (Os-Pd 2.840(1) Å) and a square planar coordination geometry around the Pd centre with a P-Pd-P angle of 165° [46].

The catalytic activity of cationic Pd(II) complexes with ligands **7**–**10** in Figure 2 is also well illustrative of how steric hindrance directs the selectivity. The catalysts with ligands **7** and **8** yield exclusively PK or MP, respectively, the ones with ligands **9** and **10** are ineffective [71].

The dippf-, dppomf- and dppo-based catalysts are moderately active (TOF_MP_ = 300–400 h^−1^ under typical carbonylation conditions (85 °C, 40 bar, CO:ethane = 1:1). A much higher performant catalyst is the one with the sterically hindered ligand **11** in Figure 2 [72]. With an excess of ligand and ethene (Pd:ligand = 1:5.3, ethane:CO = 9:1) at 100 °C and 10 bar, typical TON were 59,000–64,000, with initial TOF of 30,000–31,000 h^−1^ [73]. With this ligand there is apparently no Fe-Pd interaction, the selectivity to MP is due to the steric hindrance, as it is in the case of the catalyst based on ligand **11** in Figure 1, which is also highly active (see below).

Thus, experimental evidence points to the fact that steric hindrance plays a key role in governing the selectivity of the carbonylation of ethene. On one extreme, by carefully increasing the steric hindrance of catalysts suitable for the formation of PKs, the productivity increases, due to the destabilization of resting states preceding the insertion of the olefin, thus easing the rate determining step. Further increase of the steric bulk inhibits more and more the insertion of the olefin, to the extreme case in which after the insertion of just one molecule of each monomer, fast methanolysis of the Pd-acyl intermediate occurs with selective formation of MP as in the case of the dtbpx based-catalyst. The factors governing the selectivity toward either MP or PKs were investigated by theoretical studies for a palladium catalyst with the electro-donating 1,2-*bis*(dimethylphosphino)ethane (dmpe) ligand. It was found that the rate determining step for chain growing is the insertion of ethene into the Pd-acyl bond with formation of a β-chelate, due to the low affinity of ethene for coordination in the intermediate [Pd(COCH_3_)(ethene)(dmpe)]. For the competing methanolysis termination, the most likely route involves the formation of [Pd(CH_3_OH)(COCH_3_)(dmpe)], followed by a proton-transfer/reductive-elimination process mediated by the solvent. The overall barrier for this process is higher than that for ethene insertion, in line with the fact that this catalyst promotes the formation of a PK. Electronic bite angle effects on the rates of ethene insertion and ethanoyl methanolysis were estimated with catalysts with four electronically and sterically related ligands, (Me)_2_P(CH_2_)_n_P(Me)_2_ (n = 1–4). Steric effects were studied for larger *^t^*Bu substituted ligands. It was found that ethene coordination and subsequent insertion into the Pd-ethanoyl bond are disfavoured by increasing the steric bulk and that the key intermediates in the methanolysis step are stabilized because of electronic effects caused by increasing the bite angle of the ligand. The combined effects explain which ligands favour the formation of PKs over that of MP [40].

## 4. Highly Active and Selective Catalysts for the Ethene Hydromethoxycarbonylation

**Figure 5:**
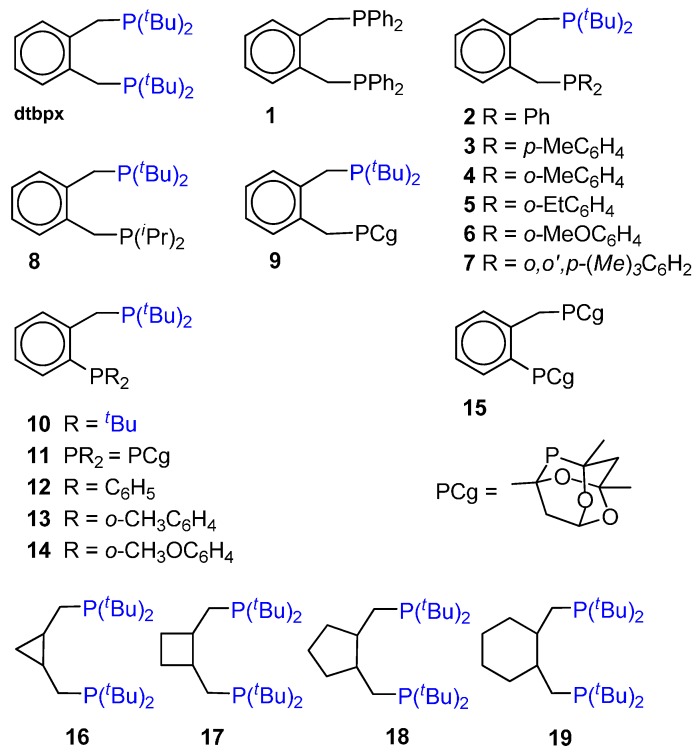
*^t^*Bu- and admantyl-ligands for the highly active (dtbpx and ligands **2**–**11** and **15**) and selective (dtbpx and ligands **2**–**11** and **15**–**19**) hydromethoxycarbonylation of ethene.

The high performance of the catalyst based on the dtbpx ligand **11** in Figure 1, used in the “Lucite” Process, prompted much interest in developing other possibly more efficient catalysts. The complexes with ligand **1** and unsymmetrical ligand **2** of Figure 5, which do not differ for the bite angle with respect to the “Lucite” ligand, show a significantly different selectivity toward MP. The one with one P(*^t^*Bu)_2_ bulky group is highly active and selective like the dtbpx catalyst, whereas with the other ligand, which has a significantly smaller steric hindrance, activity and selectivity are significantly lower (200% and 10%, respectively), the main products being oligomers (22%) and PKs (68%). It was concluded that only one P(*^t^*Bu)_2_ group is required to achieve excellent selectivity and activity and that a Pd-acyl intermediate with a *trans* P(*^t^*Bu)_2_ group dictates the chemoselective determining step (see below at point 5.1.). Quite interestingly, it was also found that the TON with ligand **2** remains high for a longer reaction time compared to the dtbpx catalyst [74].

Catalysts based on other unsymmetrical diphosphines were later developed (Figure 5, ligands **3**–**9**). All of them are very efficient, including the one with ligand **9** with the electron poor phospha-adamantyl donors, which suggests that ligand steric effects are more important than electronic ones [75].

The interplay of bite angle and cone angle effects was studied by comparing the catalytic activity using the unsymmetrical ligands **10**–**15** of Figure 5 [76]. These ligands present a smaller bite angle than **1**–**9**. Not only, they differ also for the nature of the PR_2_. The ones with bulkier PR_2_ are very active, (ligands **10**, **11** and **15**), the last two being 3.8 more active than “Lucite” catalyst. The others show zero activity. A comparative investigation of the coordination chemistry with Pd and Pt complexes suggests that, with the less bulky PR_2_, the smaller bite angle reduces the capacity of promoting the insertions or reductive eliminations steps.

Under the conditions reported in [77], the catalytic activity of the cationic complexes with ligands **17**–**19** in Figure 5, though higher than the one with the “Lucite” dtbpx ligand (TON *ca*. 850 *versus* 603, Pd:L:TFA = 1:3:125 (TFA = trifluoro acetic acid), at 120 °C, under P_ethene_ = 20 bar, P_CO_ = 45 bar, solvent MP:MeOH = 1.8:1.2 (v/v)), is much lower than the one obtained with the “Lucite” dtbpx catalyst under milder temperature and pressure conditions (TON = 17,900, at 100 °C, under P_ethene_ = Pco = 5 bar, Pd:L:MsOH = 1:5:450 (MsOH = methanesulfonic acid), solvent MeOH) [74,75,76]. The different catalytic activity with the ligands **16**–**19** was not attributed to the bite angle of the ligands that are very close for all complexes and to the difference in the rigidity of the ligand backbone. The combination of the basicity of the ligand and the strength of the acid used, however, may be related to the catalyst activity. In fact, these complexes are active if used in combination with weaker TFA acid, differently from the ones with dtbpx or with ligands **2**–**15**, which are efficient also when using the stronger MsOH acid. This acid protonates the ligands, causing decomposition to inactive Pd black [77]. The catalytic cycles of these (P-P) catalysts are discussed hereafter.

## 5. Mechanism of the Ethene Hydromethoxycarbonylation

MP may be considered the lightest member of a copolymerization process. Whereas the formation of the copolymer occurs via both the Pd-H and Pd-OCH_3_ initiators, it will be shown that in the formation of MP the Pd-H route is dominant if not exclusive.

### 5.1. Diphosphine Catalysts

Due to its importance, the catalytic cycle using the very active and selective dtbpx-based system (“Lucite” catalyst) has been studied in many details [62,63,64,65]. For this catalyst, it has been unambiguously proven that catalysis takes place via the Pd-H route. The initiator [PdH(MeOH)(dtbpx)](TfO) can be easily prepared by dissolving [Pd(TfO)_2_(dtbpx)] in MeOH [65] or another primary or secondary alcohol. This hydride is rather stable in MeOH, and deprotonation does not occur, probably because of the basicity of the ligand makes the palladium centre more nucleophilic. Though very reactive, the hydride is formed quantitatively, so that its concentration is the highest possible, which is ideal for efficient catalysis. At room temperature it reacts immediately with ethene, giving a cationic ethyl complex [Pd(Et)(dtbpx)](TfO) in which there is a β-agostic C-H interaction with the fourth coordination site. No further incorporation of ethene occurs. In THF solution, the ethyl complex reacts immediately with CO, yielding the acyl complex [Pd(COEt)(dtbpx)(THF)] and, upon adding trace amounts of MeOH, immediate formation of MP and quantitative regeneration of the hydride occurs. In MeOH the ethyl complex reacts immediately even at a low temperature with CO, yielding MP and the hydride, presumably via an acyl intermediate. Whereas with CO alone the hydride in MeOH is unstable, with both CO and ethene, decomposition does not occur. Instead, there is formation of a Pd-acyl species, which in turn reacts with MeOH with quantitative regeneration of the initiator and MP [63]. In the acyl there is no β-agostic C-H interaction and the two P atoms are equivalent even below room temperature via movement of the intact acyl group. Even when the solvent has weakly coordinating ability such as THF, the acyl complex prefers solvent coordination, rather than CO coordination, even in the presence of free CO. This can also contribute to the high activity observed for these systems, as MeOH has not to compete with CO for coordination before the product-forming step [63].

**Scheme 6:**
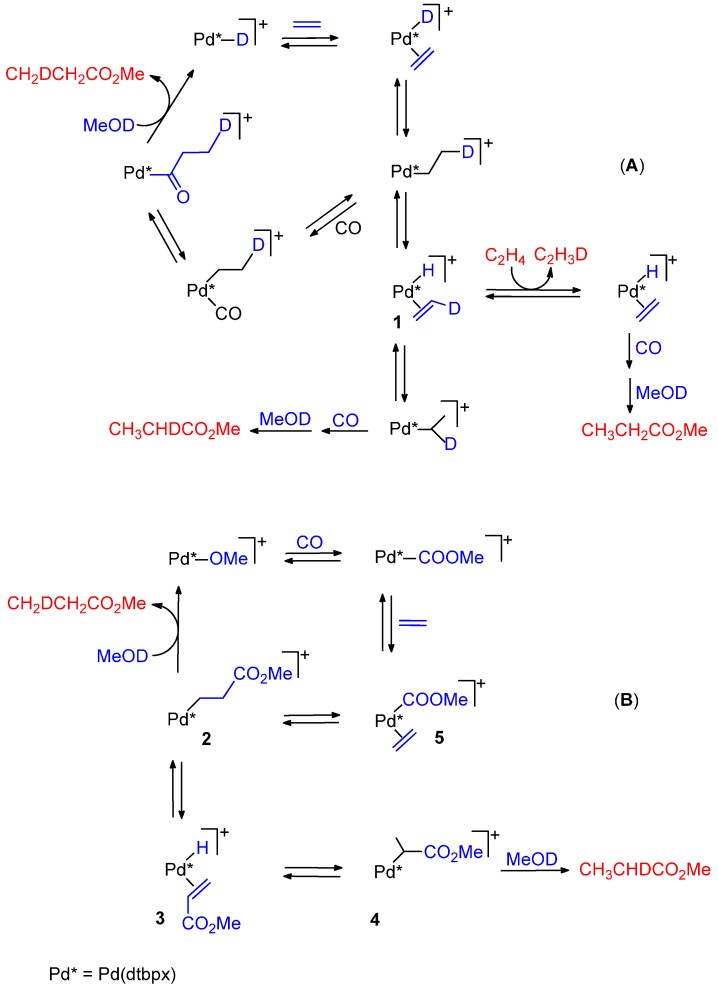
Catalytic cycle for the hydromethoxycarbonylation of ethene catalysed by the dtbpx-based system in MeOH (**A**) and in MeOD (**B**).

Further evidence in favour of the hydride mechanism that excludes the other route is provided by carrying out the catalysis in CH_3_OD under conditions of chemical or CO g/L mass-transfer control of the reaction [64]. In the first case a mixture of monodeuterated products CH_2_DCH_2_CO_2_Me and CH_3_CHDCO_2_Me in approximately 1:1 ratio is formed, together with low levels of undeuterated MP and of CH_2_DCHDCO_2_Me, with no H incorporated into the CH_3_OD. This can be explained if a reversible ethene insertion and coordination of CO occur at a much higher rate than the exchange of Pd-H with CH_3_OD. Coordination of ethene is essentially irreversible, since otherwise significant amounts of undeuterate MP would be formed from loss of C_2_H_3_D from intermediate **1**, followed by coordination-insertion of ethene into the Pd-H bond. Under conditions of CO transfer limitation, multiple labelled products would arise from Pd-H exchange with CH_3_OD in intermediate **1** (Scheme 6A). The formation of monodeuterated MP can be explained also by a “carbomethoxy” mechanism if, after migratory insertion of ethene into the Pd-COOMe bond, β-H abstraction occurs in intermediate **2** to give **3** (Scheme 6B). Deuteration of **2** and **4** by MeOD would then lead to the two products observed. Again, the termination, relative to Pd-H/CH_3_OD exchange, must be rapid, otherwise multiple deuterated products would form. Under conditions of CO starvation these products would arise from the reversible exchange between intermediates **2**, **3**, and **4**, through exchange of Pd-H with MeOD in **3**. However, this mechanism cannot explain the formation of a large amount of undeuterated MP, which is formed in the early stages of the reaction since the termination step must always transfer a D atom from CH_3_OD to end up on one of the ethyl carbon atoms of MP. It was concluded that the hydromethoxycarbonylation of ethene occurs by a hydride route in which the rate determining step comes after the insertion of ethene into the Pd-H bond and that the carbomethoxy path is not operating [64].

The fact that the carbomethoxy route is unlikely was proven in an attempt to synthesize a carboalkoxy derivative. When [PdCl_2_(dtbpx)] was allowed to react with CO in EtOH in the presence of NEt_3_, or with MeOH in the presence of Na(OCH_3_), in place of the expected carboalkoxy complex [Pd(COOR)Cl(dtbpx)] (R = Et, Me) [78,79], the Pd(0) complex [Pd(CO)(dtbpx)] was obtained [80]. Attempts to convert the carbonyl complex to a hydride by treatment with CF_3_SO_3_H failed [80].

It should be underlined that the ethyl complex does not react even with the least hindered olefin to give higher alkyl complexes or ethene oligomerization products nor the acyl intermediate inserts another molecule of ethene to give intermediates that could lead to a cooligomerization process, not even in the presence of a large excess of acid (Pd:MsOH = 1:450) [74,75,76], in contrast with the hydroacylation of ethene catalysed by a system based on ligand **13** in Figure 1, in which case the selectivity to DEK changes from 98% to 33%, the remaining being cooligomers (*ca*. 60%) [42]. The unique chemistry of the dtbpx- and ligands **2**–**11** and **15** -based catalysts is most likely due to their highly restrictive steric demand. However, the hydride catalyses the isomerization of higher 1-olefins to an equilibrium mixture of all the possible internal isomers and also the hydromethoxycarbonylation of 1-olefins and of internal olefins to the linear ester with 99% regioselectivity [81]. 

Using the precursors with ligands **16**–**19**, no Pd-H species could be detected. Indirect evidence in favour of the Pd-H path comes from the reaction with ethene in MeOH, which gives Pd-Et complexes, stable in the presence of an overpressure of ethene [77].

**Scheme 7:**
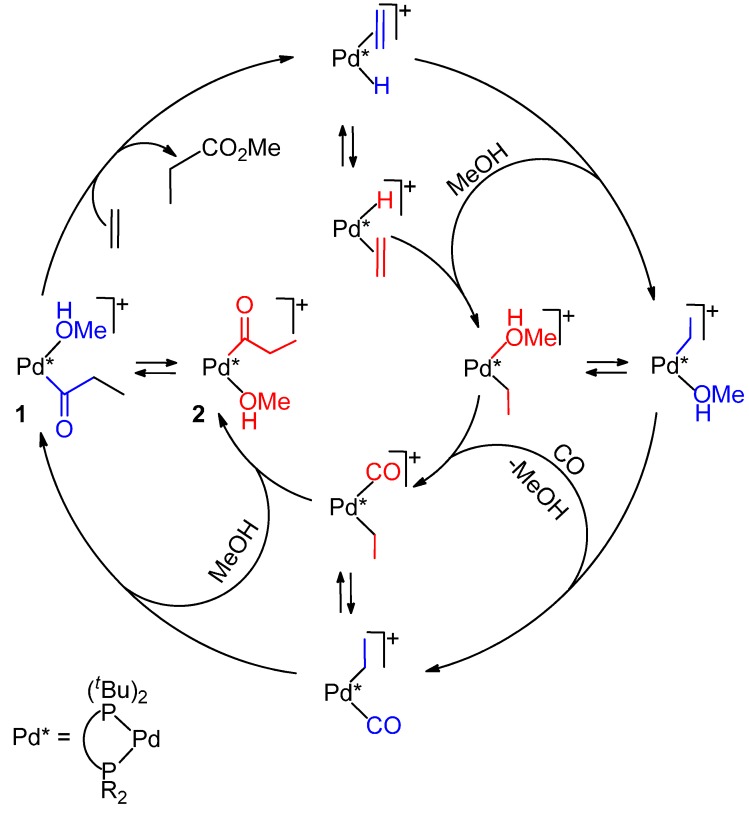
Catalytic cycle for the hydromethoxycarbonylation of ethene catalysed by complexes with unsymmetrical *^t^*Bu-diphosphine ligands.

Catalysis with the highly active and selective complexes with the unsymmetrical ligands, having one P(*^t^*Bu)_2_ group involves two geometric isomers for each of the catalytic intermediates in equilibrium as shown in Scheme 7. Since they are highly selective, it is likely that intermediate **1**, with the Pd-acyl moiety *trans* to the P(*^t^*Bu)_2_ group is kinetically dominant over diastereoisomer **2** and dictates the chemoselective product forming step [74,75,76].

Using the cationic precursors with ligands **16**–**19** of Figure 5 no Pd-H species could be detected. Indirect evidence in favour of the Pd-H path comes from the reaction with ethene in MeOH, which gives Pd-Et complexes, stable in the presence of an overpressure of ethene [77].

### 5.2. Monophosphine Catalysts

Also with monophosphine catalysts, most of the experimental evidence is in favour of the Pd-H mechanism. Using the catalytic system [Pd(TsO)_2_(PPh_3_)_2_]/PPh_3_ in ethene hydromethoxycarbonylation, it has been found that a source of hydride, such as H_2_O, TsOH or H_2_, enhances the catalytic activity [82]. Moreover, after catalysis, the acyl complex *trans*-[Pd(COEt)(TsO)(PPh_3_)_2_], relevant to the hydride route, has been isolated [83]. This complex reacts with MeOH to yield the expected MP almost quantitatively and catalyses the carbonylation of a different olefin yielding the expected ester and MP, the latter in an almost stoichiometric yield. This proves that the acyl complex is stable enough to be isolated while being reactive enough to enter the catalytic cycle [83].

The carbomethoxy complex *trans*-[Pd(COOMe)(TsO)(PPh_3_)_2_] [84] relevant to the Pd-COOMe route [85,86,87], does not insert ethene at a temperature up to 50 °C [88]. At higher temperature and in combination with PPh_3_ and TsOH, and even better in the presence of some water, catalysis is observed, however, the acyl complex *trans*-[Pd(COEt)(TsO)(PPh_3_)_2_] has been recovered after cooling [83]. Moreover, formation of dimethyl succinate was not observed. All these findings are in favour of the Pd-H mechanism rather than the other one.

Other evidence in favour of the Pd-H route has been found using the precursor *cis*-[Pd(SO_4_)(PPh_3_)_2_], which turns into an active catalyst when used in combination with H_2_SO_4_ and PPh_3_. After catalysis and upon addition of LiCl, *trans-*[Pd(COEt)Cl(PPh_3_)_2_], related to the Pd-H catalytic cycle, has been isolated. Moreover, it has been found that *cis*-[Pd(SO_4_)(PPh_3_)_2_], dissolved in CD_2_Cl_2_/MeOH in a NMR tube, reacts with CO to give a Pd-COOMe complex, which neither inserts ethene, nor gives MP. In the presence of H_2_O and H_2_SO_4_ the carbomethoxy complex is unstable giving a Pd-H complex, which promotes catalysis to MP in the presence of CO and ethene [89].

NMR studies have provided further evidence in favour of the “hydride” mechanism [90]. The reactivity of *cis*-[Pd(H_2_O)_2_(PPh_3_)_2_](TsO)_2_·2(H_2_O) (**I**), *tran*s-[Pd(COEt)(TsO)(PPh_3_)_2_] (**II**) and *trans*-[Pd(COOMe)(TsO)(PPh_3_)_2_] (**III**) has been studied by ^1^H and ^31^P{^1^H} NMR spectroscopy conditions that mime the catalytic ethene hydromethoxycarbonylation, *i.e.*, in the presence of PPh_3_, H_2_O and TsOH. (**I**), in combination with two equivalents of PPh_3_, reacts with MeOH and CO (3 bar, −80 °C) giving [Pd(COOMe)(TsO)(PPh_3_)_3_] (**III'**), which reacts at 20 °C with H_2_O in the presence of TsOH generating [PdH(PPh_3_)_3_](TsO) (**IV**) quantitatively. This hydride inserts ethene (3 bar, 20 °C) yielding *trans*-[Pd(Et)(TsO)(PPh_3_)_2_] (**V**), which reacts with CO (3 bar, −50 °C) with formation of [Pd(COEt)(PPh_3_)_3_](TsO) (**II'**) and initiates catalysis to MP at 20 °C. **II**, in combination with PPh_3_ and TsOH, reacts at 20 °C with MeOH with quantitative formation of MP and **IV** and promotes catalysis starting from this temperature, under 6 bar of CO/ethene (1:1). In the absence of added PPh_3_, MP is formed in a stoichiometric amount, catalysis is not observed and decomposition to Pd metal occurs. Therefore, PPh_3_ is essential in order to stabilize hydride **IV**. **III** does not insert ethene even at 70 °C, a temperature well above the one at which catalysis occurs. All these experimental findings are in favour of the Pd-H route [90].

Using the catalytic system [Pd(AcO)_2_(PPh_3_)_2_]/PPh_3_/TsOH, phosphine degradation side reactions with formation of phosphonium cations such as EtPPh_3_^+^ and EtCOCH_2_CH_2_PPh_3_^+^, provide further evidence in favour of the hydride path [91].

**Scheme 8:**
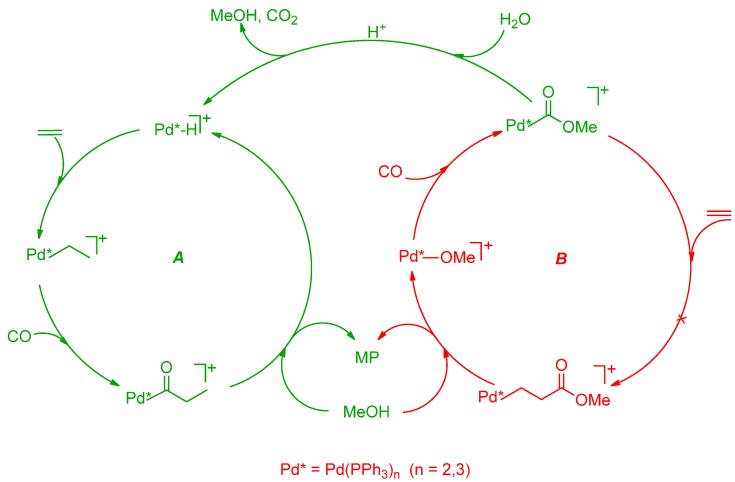
Pd-H catalytic cycle with PPh_3_-based systems (**A**); alternative (unlikely) Pd-OMe route (**B**).

In conclusion, catalysis is effective under conditions that favour the formation of a Pd-H initiator (Scheme 8) and disfavour the formation of the other possible initiator. In any case, the latter is rather reluctant to insert ethene into the Pd-COOMe bond (even in the absence of PPh_3_ that could impede the easy accessibility of ethene to the metal centre) a key step in the Pd-COOMe mechanism.

In contrast, in diphosphino complexes the insertion of ethene into a Pd-COOMe bond occurs easily. For example, [Pd(COOMe)(L)(P-P)](TfO) (L = CO, CH_3_CN); P-P = dppp, dibpp), the insertion of ethene into the Pd-COOMe bond occurs at a temperature as low as −30 °C under 1 bar of ethene, yielding a β-chelate, which reacts with MeOH at r.t. yielding MP, thus proving that with (P-P) complexes catalysis to MP may also occur via Pd-OCH_3_ route (Scheme 5C, cycle **1' → 2'→ 3' → 1'**) [34,35].

It has been shown that hydroalkoxycarbonylation also proceeds through a Pd-H mechanism using the neutral precursor [PdCl_2_(PPh_3_)_2_], with which *trans*-[PdCl(COR)(PPh_3_)_2_] and *trans*-[PdCl(COOR')(PPh_3_)_2_] have been isolated after catalysis [92,93,94]. The hydroalkoxycarbonylation of higher olefins, styrenes, monoterpenes catalysed by Pd/PPh_3_ systems also ocurrs via the Pd-H mechanism [4,93,94,95,96,97,98,99,100,101,102,103,104].

**Scheme 9:**
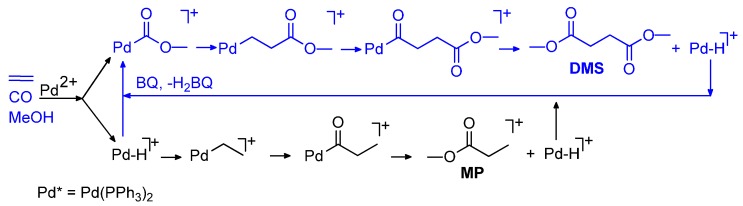
Oxidative carbonylation of ethene in the presence of BQ.

Further studies have demonstrated that ethene insertion into a Pd-COOMe bond may occur if catalysis is carried out in the presence of BQ [105] However, in this case the main products are dimethyl succinate (DMS) and dimethyl oxalate (DMO), which are formed together with minor amounts of MP and dimethyl carbonate (DMC). The formation of DMS unambiguously proves the ethene insertion into a Pd-COOMe bond. Model reactions of a Pd-hydride with BQ and of *trans*-[Pd(COOMe)(TsO)(PPh_3_)_2_] with ethene in the presence of BQ have been studied by ^31^P{^1^H} NMR. BQ consumes the Pd-hydride and directs the catalysis toward a Pd-COOMe initiator leading to DMS (Scheme 9).

It is worth noticing that catalysis occurs at 55 °C, a temperature at which the insertion of ethene into a Pd-COOMe moiety does not occur in the absence of BQ. Thus BQ, not only is the oxidant that makes the catalysis to DMS, DMO and DMC possible, but also modifies the reactivity of the Pd-COOMe moiety. In fact, *trans*-[PdCl(COOMe)(PPh_3_)_2_], used as catalyst precursor, is stable in the absence of BQ. As a matter of fact, it can be synthesized in a high yield by carbonylation of *trans*-[PdCl_2_(PPh_3_)_2_] in MeOH even at 70 °C [106].

## 6. Carbonylation in Aqueous Solvents

Replacement of volatile toxic organic solvents used in synthesis as become a more and more pressing issue for the chemical industry. Particularly in homogeneous catalysis, efficient recycling of the catalyst is needed for developing eco-friendly processes. The use of aqueous biphasic media or ionic liquids (ILs) may be a convenient solution to both the solvent emission and the catalyst recycling problem. Here below, carbonylation in water or in aqueous solvent mixtures will be reviewed. The carbonylation in ILs will also be presented briefly.

### 6.1. CO-Ethene Copolymerization with Water-Soluble Catalysts

**Figure 6:**
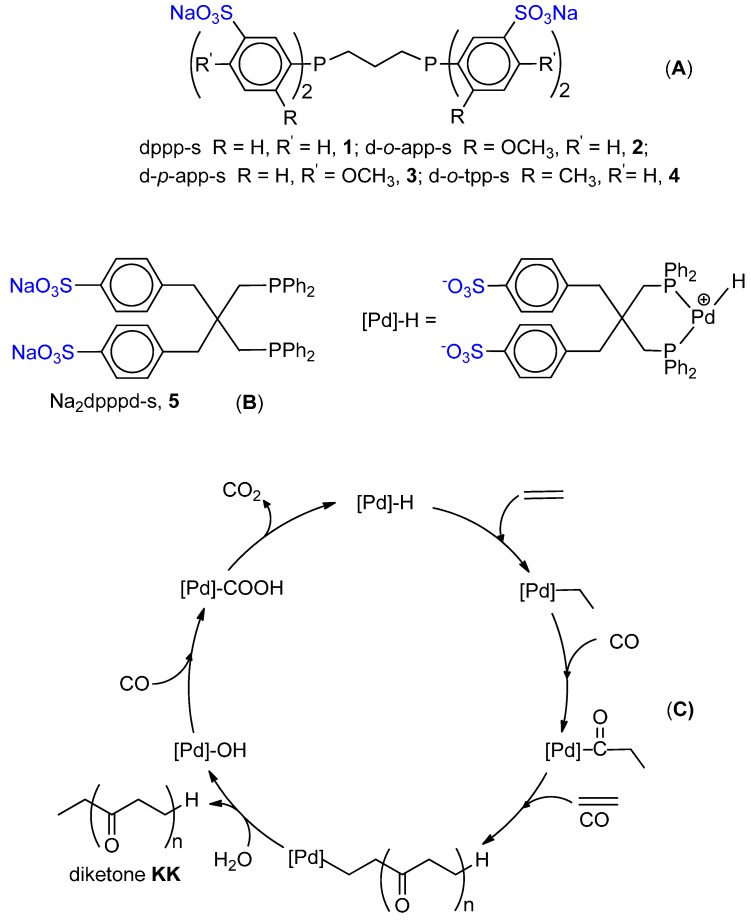
Sulfonate ligands for water-soluble catalysts (**A**); catalytic cycle for the CO-ethene copolymerization catalysed by the Na_2_dpppd-s-based system (**B**,**C**).

The CO-ethene copolymerization was first carried out by Sen *et al.* using the cationic water soluble Pd(II) catalyst with the sulfonate analogous of dppp, dppp-s (Figure 6A, K in place of Na). The activity was rather low (470 g of PK per g of Pd in 22 h, at 50 °C, under 70 bar (CO/ethene = 1/1)) [107]. A much higher productivity, close to that of the dppp system in MeOH, was achieved by Bianchini *et al.* using a Na_2_dpppd-s-based catalyst (Figure 6B) [108]. The PK has exclusively keto-end groups. *In situ* HPNMR investigations showed that the only detected complex has the ligand and TsO^−^ or H_2_O coordinated to Pd. It was suggested that this complex acts as a reservoir of “(P-P)Pd(II)” moieties which are delivered into the catalytic cycle as Pd-H species. The proposed catalytic cycle is depicted in Figure 6C.

The dppp-s catalyst in combination with TsOH (Pd(II):dppp-s:TsOH = 1:1:30) was moderately active under standard conditions (4 kg PK(g·Pd·h)^−1^ at 90 °C, 4 MPa, CO:ethene = 1:1). It is worth noticing that the ^13^C-NMR analysis showed that the perfectly alternating PK presented approximately 85% keto-end groups and 15% acid-end groups [109].

Other water-soluble catalysts with dppe-s, dpppen-s and dppbu-s, having C_2_H_4_, 2,4-*n*-C_5_H_10_ or *n*-C_4_H_8_ bridging the two P atoms, respectively, have also been tested. The first two exhibit a lower productivity of a lower molecular weight PK, whereas the other is more active that the dppp-s catalyst. The dppp-s catalyst afforded a PK of somewhat higher MW than the dppp one in MeOH [110].

Remarkable results were obtained using the precursor [Pd(CH_3_)(CH_3_CN)(d-*o*-app-s)](TfO) with the sulfonate analog of d-*o*-app having an *o*-MeO substituent group in each of the phenyl rings (32.2 kg PK(g·Pd·h)^−1^ at 90 °C, 60 bar, CO:ethene = 1:1) [111], which is four times more than the one obtainable with the dppp-s analogous). The *para*-OMe isomer, d-*p*-app-s was significantly less active than the d-*o*-tpp-s one, suggesting that the promoter effect is steric in nature. However, the d-*o*-tpp-s based catalyst, having an *o*-Me substituent group, was also considerably less active, suggesting that the effect of the *o*-MeO substituent is not only steric. The coordination of the oxygen atom was found in the solid state in [Pd{P(C_6_H_2_-2,4,6-(OMe)_3_}_2_](BF_4_)_2_ [112]. However, a coordinative interaction with the oxygen atom was not observed in the NMR spectra of the d-*o-*app-s catalytic system [111].

The d-*o-*app-s catalyst, supported on the PK itself, is also active (21.3 kg PK(g·Pd·h)^−1^. Such a “gas-phase process” reduces at a minimum the volume of the solvent and avoids fouling drawback [111].

A similar catalyst, prepared from ligand d-*o*-app-s, Pd(AcO)_2_ and TFA acid (ligand:Pd:TFA = 1.1:1:4), was used for the CO-ethene-propene terpolymerization in the presence of H_2_, in H_2_O-MeOH-AcOH-MeOAc (29.5:45.4:5.3:19.6 v/v) as a solvent [60]. AcOH stabilizes the catalyst, and water improves the product-solvent separation. Viscous low molecular weight ter-oligomers were obtained in high yield (9.9 kg PK(g·Pd·h)^−1^, with 25%–35% of propene in the 1,3-mode, after 3 h at 91 °C, H_2_:ethene:propene:CO = 7:5:30:21).

The catalysts with ligands **2** and **3** of Figure 2 show some activity in H_2_O if used in combination with TsOH. In H_2_O:dioxane = 1:1 (v/v), the productivity of the catalyst with ligand **3** significantly improves when used with TsOH, BQ or a combination of them (11.2 kg PK(g·Pd·h)^−1^ Pd:BQ:TsOH = 1:80:20, at 85 °C, 40 bar, CO:ethene = 1:1). They are active also for the terpolymerization of CO with ethene and propene in H_2_O/1,4-dioxane [69].

### 6.2. Monocarbonylation of Olefins

The use of water soluble catalysts for the carbonylation of olefins was first reported by Seldon and Monflier [113,114]. The catalyst, prepared *in situ* from PdCl_2_ and tppt-s (tppt-s = P(C_6_H_4_-*m*-SO_3_Na)_3_) in combination with excess TsOH or TFA, was exceptionally highly active for the hydroxycarbonylation of propene (TOF > 2500 h^−1^ at 120 °C, 50 bar) [113].

**Scheme 10:**
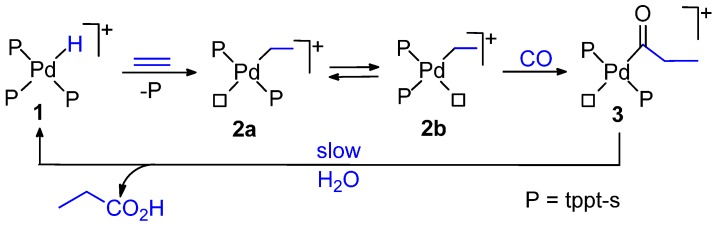
Catalytic cycle for the carbonylation of ethene in H_2_O.

The carbonylation of ethene to propionic acid was studied using the water-soluble catalyst prepared *in situ* from Pd(AcO)_2_ and tppt-s in ratio 1:4 in 60% (v/v) aqueous TFA. Pd-hydride, Pd-ethyl and Pd-acyl species were detected by NMR spectroscopy. Kinetic investigations showed that the slow step is the hydrolysis of the Pd-acyl complex to the product forming step. Scheme 10 shows the proposed catalytic cycle. In addition to propionic acid, there was formation of minor amounts of the co-oligomer keto acids, CH_3_CH_2_(COCH_2_CH_2_)_n_COOH (n = 1–3) [115].

**Figure 7:**
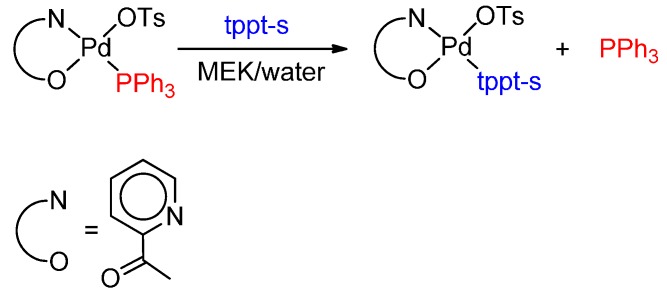
Water-soluble catalyst precursor with tppt-s ligand.

Because of their low solubility in water, the carbonylation of higher olefins, such as styrene derivatives or higher 1-olefins, occurs in low yield and, to be efficient, requires the use of mass transfer promoters such chemically modified cyclodextrins [114]. The necessity of using a mass transfer agent is avoided by using the water soluble precursor shown in Figure 7, in combination with LiCl and TsOH, which is highly active and selective in the carbonylation of vinyl aromatic compounds dissolved in toluene (TOF = 282 h^−1^, selectivity toward the *iso*-acid 91%, at 110 °C, 54 bar) [116].

### 6.3. Copolymerization in Water-Organic Acid

The CO-ethene copolymerization in water-acetic acid was first reported by Toniolo *et al.* using the [PdX_2_(dppp)] (X = AcO, Cl) precursors, which are inactive in MeOH. Under standard conditions, an exceptionally high productivity was achieved (28 kg PK(g·Pd·h)^−1^), which is the highest up to now reported in this solvent [52,117].

**Scheme 11:**
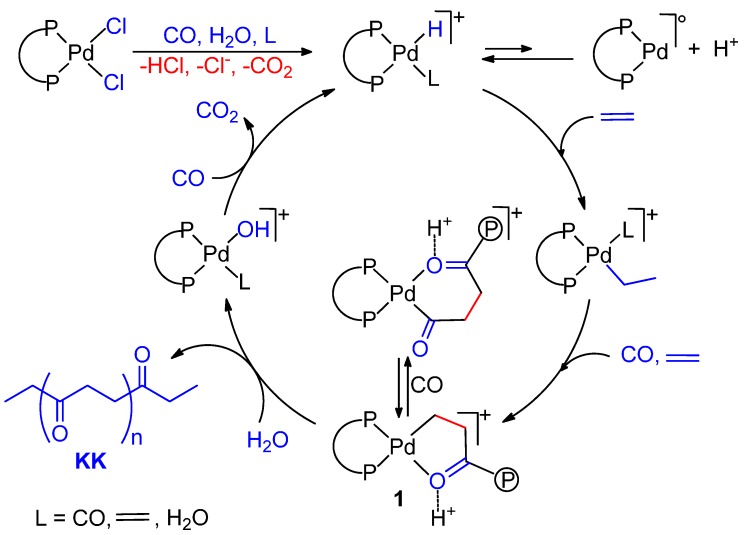
Activation of the precursor [PdCl_2_(P-P)] and catalytic cycle for the copolymerization of ethene in H_2_O-organic acid as a solvent.

^13^C-NMR analyses showed that the monomers are perfectly alternating even when one monomer is used in large excess with respect to the other. Moreover, the chains end exclusively with keto groups. In addition, during catalysis CO_2_ evolution is observed. This is consistent with a chain transfer process occurring through protonolysis with H_2_O giving the PK and a Pd-OH^+^ species, which regenerates the Pd-H^+^ after insertion of CO and evolution of CO_2_. It was suggested that the Pd-H^+^ initiator may form as follows. The interaction of CO and H_2_O with the metal centre may cause the displacement of a Cl^−^ ligand, and the formation of a Pd-H^+^ species. The high pressure of the monomers, the high concentration of H_2_O and the high dielectric constant of the reaction medium also favour the dissociation of the other Pd-Cl bond, giving rise to a cationic coordinatively unsatured Pd-H^+^ species having easily available coordination sites, as when starting from a cationic precursor with weakly coordinating anions which is active in MeOH (Scheme 11).

The productivity strongly depends on the solvent composition and passes through a maximum upon increasing the acid concentration. The acid may stabilize the Pd-H^+^ species which initiates the catalysis and may destabilize the β- and γ-chelates though protonation of the oxygen atom coordinating the metal centre, thus speeding up the monomer insertions and chain growth. Water may also have a beneficial effect. Labile coordination of H_2_O may stabilize three-coordinated species, which may form during the catalysis, against their tendency to dimerize to less active species [11]. On the other hand, a too high H_2_O concentration may have a detrimental effect because of the decreasing of the solubility of the monomers [117]. 40%–60% of water seems to be the best compromise between these effects.

The use of this solvent was extended to the CO-ethene copolymerization catalysed by the precursor [Pd(SO_4_)(dppp)], which is also inactive in MeOH, unless used in combination with excess of H_2_SO_4_ (H_2_SO_4_:Pd = 1000–3000:1) [118] and the precursors [PdCl_2_(dppf)] and [PdCl_2_(dapp)] [119,120], which are also inactive in MeOH. With [PdCl_2_(dppf)] under standard conditions, up to 15.5 kg PK(g·Pd·h)^−1^ of a PK of moderately high molecular weight (Limiting Viscosity Number, LVN = 0.19 dLg^−1^) was obtained. It is worth noticing that the cationic complex [Pd(TsO)(dppf)] in MeOH is only moderately active and yields MP, DMS and low molecular weight co-oligomers [45]. Thus, the use of water-AcOH pushes the carbonylation process toward the formation of PKs. Also in this case the productivity passes through a maximum upon increasing the acid concentration. Differently, LVN increases steadily. This behaviour can be explained considering that the slow step of the chain-transfer process is the β-H elimination (*cfr*. Scheme 4A, with H_2_O in place of MeOH), which requires an available coordination site and therefore competes with any species capable of coordinating. Thus, upon increasing the acid concentration, and hence the AcO^−^ one, the enolate formation is disfavoured. Consequently, the chain growing process may go on, even though the productivity lowers. A similar explanation may also account for the observed increase of LVN upon adding NaOAc or upon increasing the pressure of CO.

The influence of the operating conditions was investigated with the precursor [PdCl_2_(dapp)]. Under standard conditions, productivity as high as 20 kg PK(g·Pd·h)^−1^ is achievable. High molecular weight PKs are produced under high pressure at a relatively low temperature, without sacrificing the productivity too much (LVN > 10 dLg^−1^, 6 kg PK(g·Pd·h)^−1^, P 110 bar, CO:ethane = 1:1, AcOH 70%) [120]. 

Other studies have been carried out using [PdCl_2_(dppf)] in water-formic acid as a solvent for the copolymerization of ethene [121] and the terpolymerization of propene and ethene with CO [122] as well as using [Pd(AcO)_2_(dppp)] in this solvent together with aprotic solvents, such as 1,4-dioxane or nitromethane [123]. In general, in H_2_O-HCOOH higher productivity and LVN are obtained with respect to the corresponding cationic catalysts in MeOH or the neutral catalysts in H_2_O-AcOH. An exceptionally high productivity was obtained in HCOOH-H_2_O-1,4-dioxane, though under higher pressure (37.5 kg PK(g·Pd·h)^−1^, LVN 2.77 dLg^−1^ at 90 °C, 90 bar, CO:ethene = 1:1, HCOOH:H_2_O:1,4-dioxane = 2.7:1.35:1) [123].

**Figure 8:**
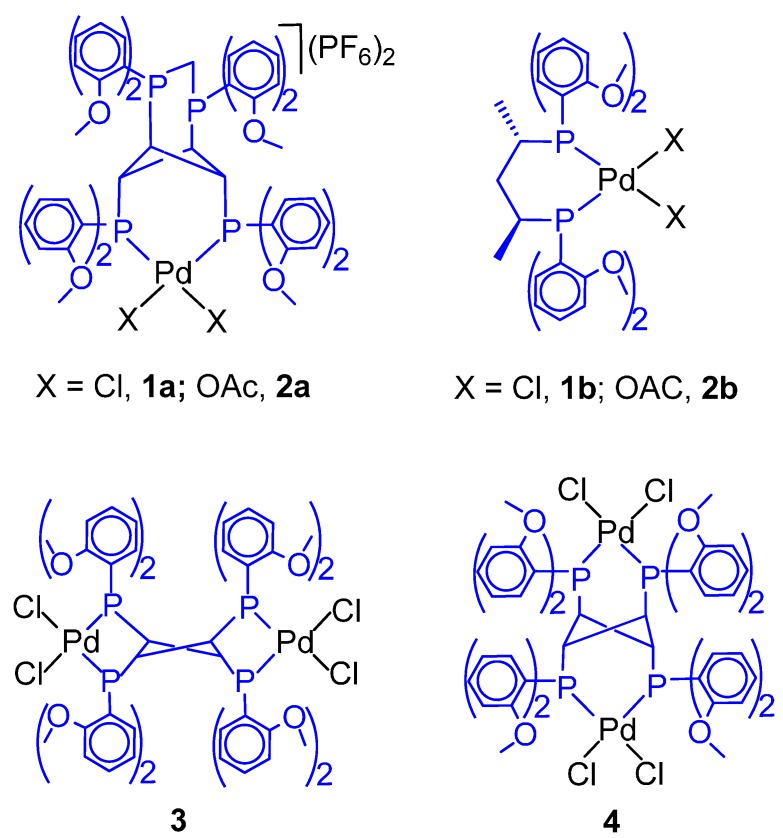
Catalysts with ligands of different stereochemical rigidity.

A comparative study was carried out using the cationic precursors [Pd(TsO)(H_2_O)(P-P)](TsO) and neutral precursors [PdCl_2_(P-P)] (P-P = dppe, dppp, *o*-MeO-dppe, *o*-MeO-dppp) in either protic or aprotic solvents. In particular, in MeOH or H_2_O-AcOH higher productivities as well as higher molecular weights were achieved for both the dppp-like catalysts with respect to the corresponding dppe-like catalysts (*i.e.*, dppp > dppe and *o*-MeO-dppp > *o*-MeO-dppe) and the *o*-MeO modified catalysts are by far more productive than their counterparts and also yield higher molecular weight PKs. Unlike the neutral complexes, the ^1^H-NMR spectrum of the cationic *o*-MeO-dppp complex indicates no interaction between palladium and the *ortho*-hydrogen atoms of the aryl groups, thus providing valuable information on the catalysis resting states and intermediates and contributing to rationalize the catalytic activity. Aprotic solvents, such as CH_2_Cl_2_ or toluene, promote the formation of high molecular weight PKs, though in unsatisfactory yields [124]. Another comparative study was performed using precursors **1a** and **1b**, in AcOH-H_2_O and **2a** and **2b** in MeOH (Figure 8) for the CO-ethene copolymerization [125].

In the latter solvent, 2 and 20 equivalents of TsOH were used as scavenger of the acetate ion. Under standard conditions, the productivity was comparable in all cases. The most striking difference between **1a** and **1b** was the M_n_, *ca.* 7 kg·mol^−1^ with **1a** and 37 kg·mol^−1^ with **1b**. In MeOH **2a** gave a lower M_n_ (8–14 kg·mol^−1^) compared to **2b** (47–48 kg·mol^−1^). Moreover, with either catalyst, higher M_n_ values were achieved in MeOH than in H_2_O-AcOH, which indicates a slower termination rate in the former solvent. The higher M_n_ obtained with **1b** and **2b** was explained taking into account the influence of a possible interaction of the *o*-methoxy moiety with the metal centre on the β-H elimination, the slow step in the chain transfer process. The stereochemical rigidity of the ligand in **1a** and **2a** would disfavour such an interaction, whereas a simple twisting of the C_5_ backbone in **1b** and **2b** would favour it, thus leading to PKs of higher M_n_.

Complexes **3** and **4** in Figure 8 were tested for the catalytic copolymerization of CO with ethene or propene and for the CO-ethene-propene terpolymerization. Their catalytic activity was compared to that of [PdCl_2_(*o*-MeO-dppe)] and [PdCl_2_(*o*-MeO-dppp)]. The highest productivity with **3** and **4** was obtained with 65 mol % and that of *o*-MeO-catalysts with 75 mol % H_2_O-AcOH. For the CO-ethene copolymerization, **3** yielded the highest productivity (12.20 kg PK(g·Pd·h)^−1^ under standard conditions), which was about three times higher than with **4** and higher than the one with the *o*-MeO-catalysts.

The different overall conformation of the ligands in the stereochemically rigid complexes **3** and **4** may be related with the different productivity. Moreover, since these complexes gave PKs of comparable M_n_, it was suggested that the metal centre in both catalysts has comparable electrophilicity and, hence, capacity to promote the β-elimination of the Pd-alkyl species. Similar trends were obtained in CO-propene copolymerization, though the productivity was about one order of size lower. In the terpolymerzation, **3** and **4** gave PKs with a significantly lower content of propene, the *o*-MeO-dppe yielded the terpolymer with the lowest melting point (150 °C, 5.51 kg PK(g·Pd·h)^−1^) [126].

It is worth reporting also here the results obtained with Pd(II)-monophosphine complexes in AcOH containing small amounts of H_2_O (1%–5%). The system Pd(acac)_2_/L (Pd:L = 1:10, L = PPh_2_(C_6_H_4_-*m*-SO_3_Na) catalyses the monocarbonylation of ethene to propionic acid at atmospheric pressure and 70 °C. Under high pressure, the reaction switches from monocarbonylation to polycarbonylation when the sodium ion is completely replaced by a proton. When the ligand is present both in the acid and the neutral form, products of di- and oligocarbonylation Et(COCH_2_CH_2_)_n_COOH (n = 1–3) are formed along with propionic acid and PK [127]. When L = PPh_3_, the system is active in the presence of large amounts of TsOH. The reaction switches from monocarbonylation to polycarbonylation at high TsOH: Pd ratio (>500) [128].

### 6.4. Biphasic Copolymerization in the Presence of an Emulsifier

The water soluble complexes [Pd(AcO)_2_(R_2_P(CH_2_)_3_PR_2_] (**a**–**h**; **a**–**g** R = (CH_2_)_n_OH, n = 1, 3–8; **h** R = (CH_2_)_3_CH(CH_2_OH)_2_, in combination with HBF_4_ (Pd:B = 1:10) are highly active in the CO/ethene copolymerization under biphasic conditions (water-toluene). In the presence of potassium dodecylsulfate as an emulsifier and MeOH as an activator, the catalytic activity increased by a factor of about three (1672 g PK(g·Pd·h)^−1^, M_w_ = 46.5 kg·mol^−1^, M_w_/M_n_ = 2.2 for catalyst **e**) under standard conditions. Also higher olefins could be successfully incorporated into the copolymerization with CO and the terpolymerization with ethene and CO. Compared to the sulfonate dppp-s catalyst, the water soluble catalyst just reported is more active in the 1-hexene incorporation in the terpolymerization of ethene with 1-hexene and CO [129]. This study has been extended to the use of catalysts with ligands of the general formula CH_2_(CH_2_PR_2_)_2_, R = (CH_2_)_n_P(O)(OEt)_2_ n = 2–6 and 8 and R = (CH_2_)_3_NH_2_ for the CO-alkene copolymerization (alkene = propene, butene or their equimolar mixtures). The catalytic activity of the dicationic complexes depends on the length of the alkyl chain. The catalyst with n = 3 is highly active for the copolymerization with propene, whereas the one with n = 6 is active for CO-butene and CO-propene-butene polymerizations. The use of β-cyclodextrin, as a phase-transfer agent, and undecanoic acid, as an emulsifier, increase the stability of the PKs and the catalytic activity [130].

Latices of CO/1-olefin PKs and CO-ethene-undec-10-enoic acid terpolymers have been prepared using Pd(II) cationic complexes with dppp or the analogous ligand with the alkyl chain (CH_2_)_13_CH_3_ in place of the phenyl ring in miniemulsion droplets of a hydrocarbon dispersed in a continuous aqueous phase in the presence of sodium dodecylsulfate (SDS) as a surfactant [131]. The productivity was comparable to that achievable with cationic dppp catalysts in MeOH. The CO-ethene copolymerization has been carried out in H_2_O/CH_2_Cl_2_ mixtures in the presence of SDS. In the presence of an excess of TsOH (TsOH:Pd = 100:1), with 20 molar % CH_2_Cl_2_ in H_2_O, 10 mM SDS, at 90 °C, 45 bar, CO:ethene = 1:1, 13 kg PK(g·Pd·h)^−1^ having LVN = 1.8 dLg^−1^ have been obtained [132].

## 7. Carbonylation in Ionic Liquids

**Figure 9:**
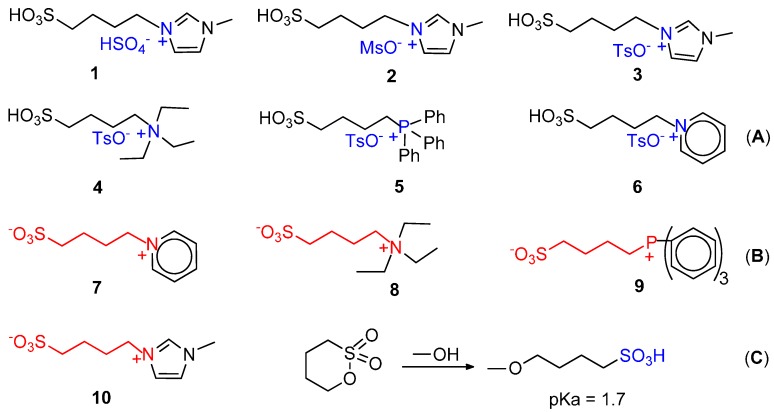
BAILs (**A**) and zwitterions (**B**) for the ethene hydromethoxycarbonylation; opening of 1,4-butansulfone with MeOH to the corresponding acid (**C**).

One of the first reports on the use of ionic liquids deals with the palladium-catalysed alkoxycarbonylation of styrene in bmimBF_4_-cyclohexane. High selectivity (≥99.5% *iso*) was obtained using [PdCl_2_(PhCN)_2_] in combination with (+)-neomenthyldiphenylphosphine and TsOH under mild conditions (70 °C, 10 bar) [133]. Very recently, the Brӧnsted acid ionic liquids (BAILs) shown in Figure 9A have been used for the hydromethoxycarbonylation of ethene.

With the system Pd(AcO)_2_/dtbpx (1/5) in MeOH with 32% of BAILs, at 80 °C, 22 bar, CO:C_2_H_4_:Ar = 2:2:1, conversions and selectivities are comparable to those obtained with the “Lucite” catalyst. BAILS serve as both acid promoters and reaction media and provide a stable biphasic system from which the product can be conveniently separated, allowing its reuse up to 15 times without apparent loss of performance [134].

It is also interesting to report the hydroalkoxycarbonylation of ethene promoted by zwitterions, which are viable feedstocks for ILs synthesis. The zwitterions shown in Figure 9B enhance the catalytic activity of a Pd(II)/PPh_3_ system, in combination with MsOH or 1,4-butansulfone, which in MeOH is opened to the corresponding acid (Figure 9C). At 100 °C, 20 bar total pressure of CO, ethene and argon in the ratios 2:2:1, with Pd(AcO)_2_, PPh_3_, **8** and sulfone in the ratios 1:10:5:60, the conversion to MP is about 98%, with selectivity ≥99%. PPh_3_ alkylation and reaction vessel corrosion are less severe [135].

## 8. Supported Copolymerization

This subject has received little attention up to now, despite its undisputable interest. The application of the d-*o-*app-s highly active catalyst in the “gas phase” CO-ethene copolymerization has been mentioned above [111]. More recently, Belov *et al.* studied the kinetics of the CO-ethene copolymerization catalysed by [Pd(TsO)_2_(dppp)] (**1**) dissolved in MeOH or immobilized on a PK as a support suspended in MeOH, toluene or heptane, in the presence of TsOH [136]. In MeOH, the rate remains nearly invariable within 1 h, whereas with the suspended catalyst the rate is higher for about the first 10 minutes, then the catalysis almost quits. It was suggested that the PK formed in the latter case encapsulates the active centres preventing further reaction. Instead, with the catalyst suspended in the hydrocarbon solvents the rate remains almost constant and is comparable to that of the homogeneous system in MeOH. In toluene the copolymer was formed as swollen mass. It is likely that the swollen PK does not impede the transport of the monomers to the palladium centres. The melting point of the PKs obtained with the supported catalysts is significantly lower, which makes the PK more easily processable.

Kinetics studies were extended to the use of high surface area SiO_2_ [137]. The activity was compared to that of the supported catalyst reported just above, using heptane/toluene (55/10, v/v) as a suspension medium. Under standard conditions, the PK yield increases up to 27 g PK(g_supported catalyst_)^−1^ upon increasing the concentration of **1** in the support up to 2·10^−4^ mol(g_supported catalyst_)^−1^ and then the yield remains practically constant, suggesting that beyond a certain concentration, the exceeding catalyst does not take part in the reaction. For the catalyst supported on SiO_2_, which has a higher specific surface area than that of the PK (250 m^2^·g^−1^ *versus* 10 m^2^·g^−1^), the PK yield increases up to a catalyst concentration of 3·10^−4^ mol(g_supported catalyst_)^−1^.

**1**/PK was also used for the terpolymerization of propene or 1-hexene with CO and ethene [138,139]. Supported [Pd(TsO)_2_(bipy)] has been studied for CO-ethene-styrene terpolymerization [140] and for the CO-cyclic olefins copolymerization [141].

## 9. Nonalternating CO-Ethene Copolymerization

The alternating CO-ethene PKs possess chemical and physical properties suitable for many applications, but suffer of low processability, due to their relatively high melting point (240–260 °C) and low solubility in common solvents. The problem has been overcome by carrying out the copolymerization in the presence of propene, which is incorporated in the PK chain, yielding a melt processable terpolymer. Another way to lower the melting point is the incorporation of extra ethene in the PK chain. The interest for copolymers having extra ethene incorporated in the linear chain is also due to the possibility that this material might be more stable than PKs with strictly alternating monomers, particularly when exposed to high temperatures for prolonged periods [142,143].

**Figure 10:**
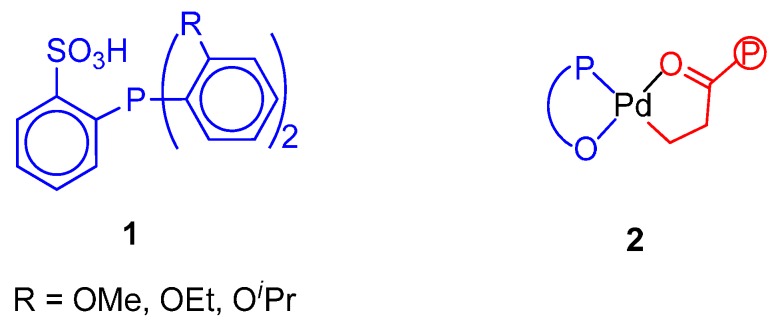
Undeprotonated ligands for the nonalternating CO-ethene copolymerization (**1**); β-chelate with the P-O^−^ ligand (**2**).

The nonalternating copolymerization has been briefly reviewed [17,18,24,144]. The first example of nonalternating copolymerization was reported by Drent [145]. The catalysts prepared *in situ* by reaction of Pd(AcO)_2_ with the undeprotonated ligands depicted in Figure 10 promote the double, triple and quadruple ethene insertions. The productivity is low compared to the Pd(P-P) cationic systems, increases with the ethene/CO ratio and with the temperature and is in the order OMe > OEt > O*^i^*Pr, which is the opposite of that of the incorporation of extra ethene. The PK produced with ligand with *^i^*Pr substituent at 110 °C, 50 bar, ethene:CO = 3:2) has 18% of non-alternating ethene insertions and melts at 229 °C. All the PKs have M_n_ > 30,000 g·mol^−1^.

The PKs do not originate from the insertion of hypothetically formed but-1-ene, as demonstrated by ethene-CO-but-1-ene terpolymerization experiments. It was suggested that the active catalyst is a neutral Pd(II) species *cis*-coordinated by a P-O^−^ chelate ligand and a five-membered β-chelate (**2** in Figure 10), in which a stereoelectronic destabilization of the palladacycle allows ethene to compete with CO for the next insertions.

**Figure 11:**
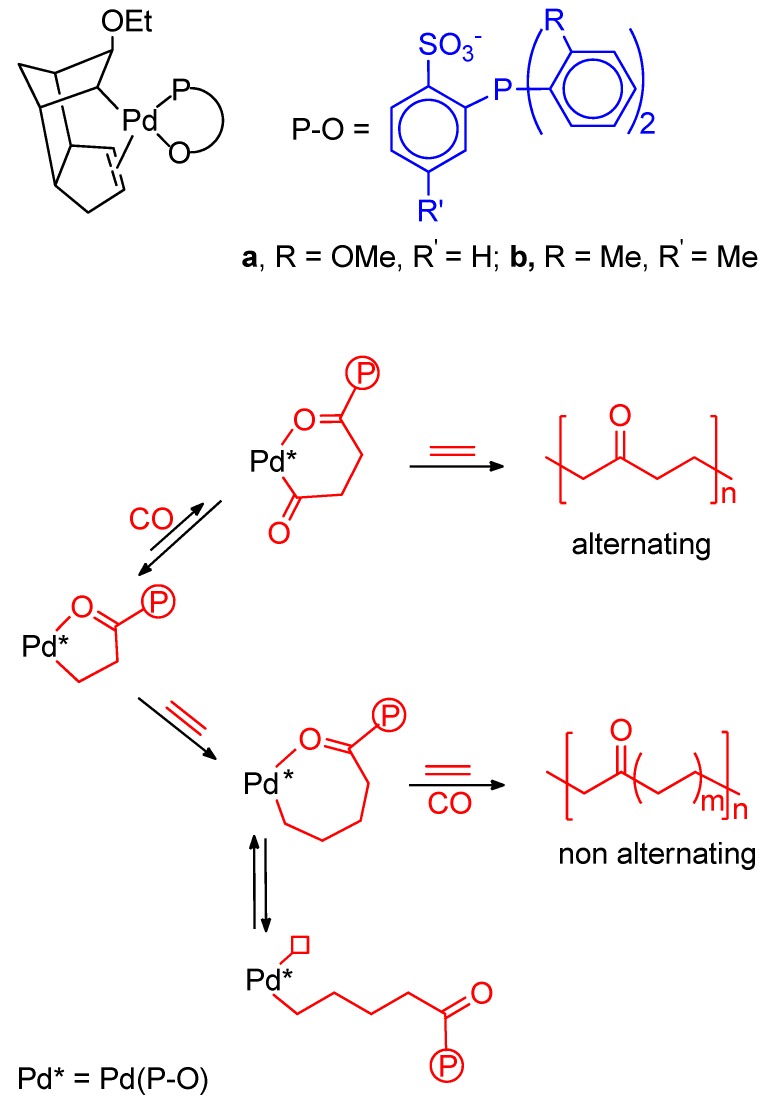
Catalysts for the CO-ethene copolymerization and alternating and nonalternating routes.

In a subsequent study, the structurally well characterized catalysts shown in Figure 11 were tested in CH_2_Cl_2_ or in MeOH [146]. The highest extra ethene insertions (29%) was obtained in MeOH at 110 °C, 65 bar, ethene:CO = 20:1. The PK presents a high M_n_ (*ca.* 370,000 g·mol^−1^) and a low melting point of 220 °C. The proposed mechanism is depicted in Figure 11, in which a β-chelate species is in common with both alternating and non-alternating routes. It was proposed that neutral P-O^−^ chelates shift the equilibrium between the six- and five-membered ring toward the latter, so that, particularly under high ethene/CO ratios, the insertion of ethene into the five-membered ring may take place, with formation of a seven-membered one, which may open more easily and allow further olefin insertion. When CO insertion into the β-chelate prevails, a perfectly alternating PK is formed.

**Figure 12:**
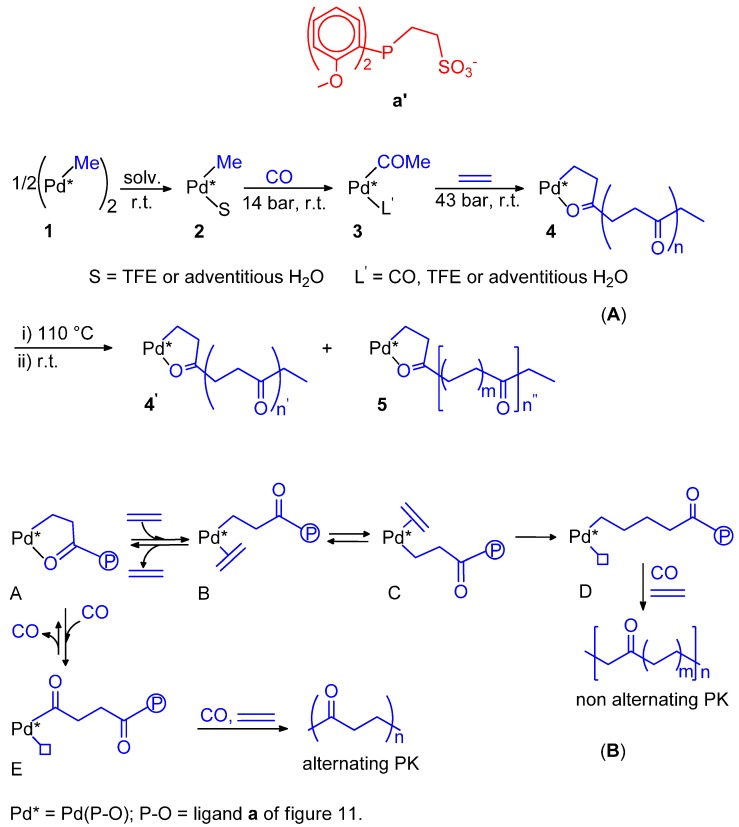
Reactivity of precursor **1** (**A**) and β-chelate opening by ethene and CO with formation of nonalternating and alternating chains, respectively (**B**).

Subsequent experimental and theoretical studies have shed more light on the key factors relevant to the non-alternating process. Bianchini *et al.* reported a comparative study of the nonalternating copolymerization in MeOH, catalysed by neutral complexes with ligand **a** and **a'** in Figure 11 and Figure 12, respectively.

The catalyst with ligand **a**, having a lower skeletal flexibility, is more active and yields a PK with a higher M_n_ and promotes higher extra-ethene incorporation. The melting point of the polymer with 27.8% of extra ethene is 193 °C. End-group analysis showed the presence of ester, ketone, vinyl end-group, in the ratio 10:10:1 [147]. *Operando* NMR experiments have been carried out starting from the model methyl complex **1** in TFE/C_6_D_6_. The results are shown in Figure 12A and can be summarized as follows. **1** is converted immediately into **2**. Under 14 bar of CO at r.t., **2** is quantitatively converted into the acyl complex **3**. After replacing CO with a ^13^C/^12^CO (1:9) mixture there was no evidence of CO coordination to the metal centre. After **3** was formed, admission of ethene (43 bar) at r.t., caused the formation of a mixture of β-chelates, **4**. Heating up to 110 °C and cooling down to r.t., the broader NMR signal was attributed to the formation of further generations of β-chelates with different alternating and nonalternating chains, **4'** and **5**.

In no case the formation of CO adducts was observed, which is believed to give a chance to ethene to insert into the β-chelate, with formation of the non-alternating chain (Figure 12B), in contrast with cationic Pd(II)(diphosphine) catalysts, which give CO adducts and yield alternating PKs. Previous theoretical studies have shown that the negligible capacity of CO to bind strongly plays a key role for favouring multiple ethene insertions and also predicted the shift from alternation to non alternation at a high temperature [148,149].

**Scheme 12:**
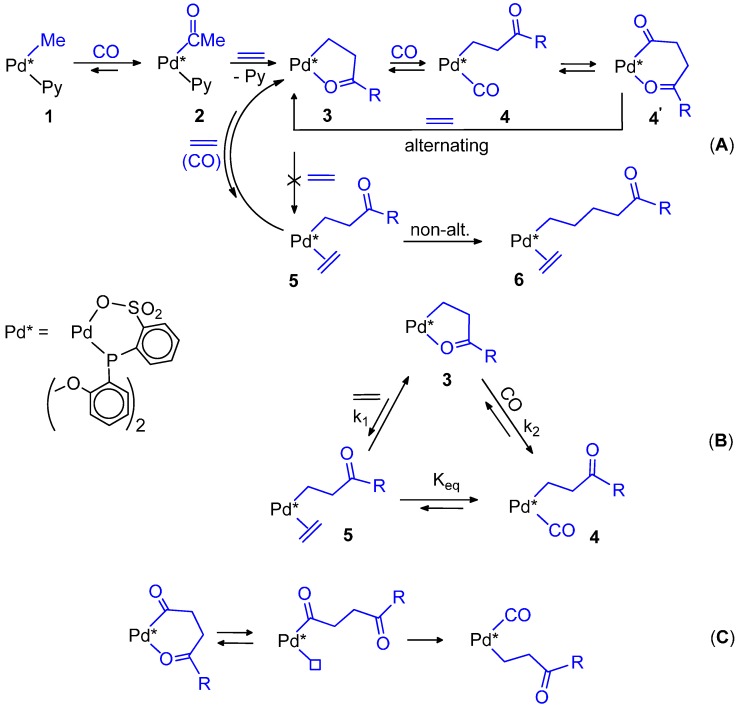
Alternating and nonalternating CO-ethene copolymerization catalysed by a (P-SO_3_^−^)-based system (**A**); β-chelate opening and competitive ethene and CO coordination (**B**); decarbonylation of a γ-chelate intermediate (**C**).

Contemporaneously, Sen also studied the stepwise insertion of the monomers starting from the model complex [Pd(Me)(Py)(P-O)] (**1**) [150]. **1** reacts with CO at room conditions, giving the corresponding acyl complex **2**. The insertion is reversible and consists of a slow decarbonylation, followed by rapid insertion, as predicted by Ziegler *et al.* [149]. **2** reacts with ethene (*ca.* 7 bar, 0 °C), to give the five-membered chelate **3**, which reacts with CO (3.5 bar, r.t.) with formation of a six-membered chelate **4**' (or its open-chain analogue **4**). This complex, exposed to ethene (7 bar at 0 °C, for 18 h, gives a five-membered species similar to **3**. **3** does not react with ethene alone (7 bar, r.t.), but, in the presence of small amounts of CO, slow consecutive ethene insertions occur at 75 °C, with formation of the nonalternating copolymer (Scheme 12A). It was suggested that step **3**→**5** does occur because the carbonyl group of the chelate **3** binds less strongly to Pd unlike in the cationic catalysts which yield the alternating PKs [149].

Subsequently, the sequence of the transformation of **3** with CO or ethene depicted in Scheme 12B was studied. At 25 °C, binding affinities of CO and ethene to the palladium chelate complex **3** are in the ratio of *ca*. 50:1 [151]. Though small, the difference in the monomer binding affinity seems to play an important role in governing the chain growing process, though, alone, it cannot explain the extent of the nonalternation actually observed. As a matter of fact, theoretical studies have found that, in contrast with the cationic (P-P) complexes, the neutral complexes with the bulky P-O^−^ ligand used by Drent do not actually form a γ-chelate six-membered ring, thus leaving a free coordination site, which may facilitate the decarbonylation of the Pd-COCH_2_CH_2_R moiety, which interrupts the alternating process (Scheme 12C). Displacement of coordinated CO by ethene would shift the copolymerization toward the nonalternation since the insertion barrier is lower [149].

**Figure 13:**
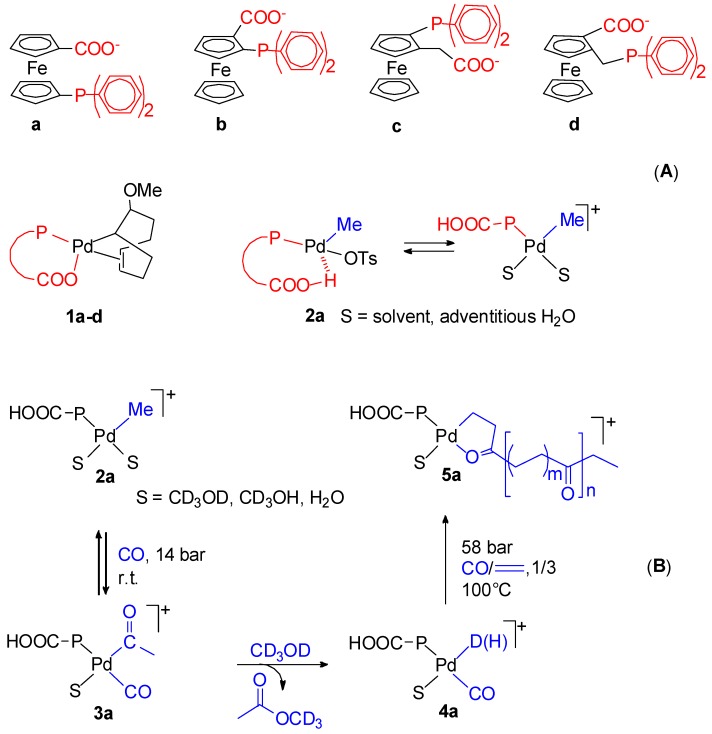
Fospharylferrocenecarboxylate ligands and relevant catalyst precursors (**A**); reactivity of a Me-model complex with CO and ethene (**B**).

Neutral Pd(II) complexes **1a**–**d** and **2a** with the phosphanylferrocenecarboxylate ligands **a**–**d** (Figure 13A), in combination with TsOH or BQ, have been tested for the carbonylation of ethene at 100–120 °C, pressure 58 bar, CO:ethene = 1:3 [152].

TsOH prevented decomposition to palladium black, BQ had a negative effect. The productivity and the incorporation of extra ethene were *ca*. one order of magnitude inferior to that of Drent catalysts, which are active even in the absence of TsOH [145]. *Operando* ^31^P{^1^H} NMR studies starting from the methyl complex **2a** in [D_4_]MeOH in the presence of TsOH allowed to detect the species shown in Figure 13B, in which **5a** is a β-chelate with growing chains of different lengths, including nonalternating ones. This species was detected after cooling the NMR tube to r.t. These results confirm that β-chelates play a key role in the nonalternating process as first proposed by Drent [145].

**Figure 14:**
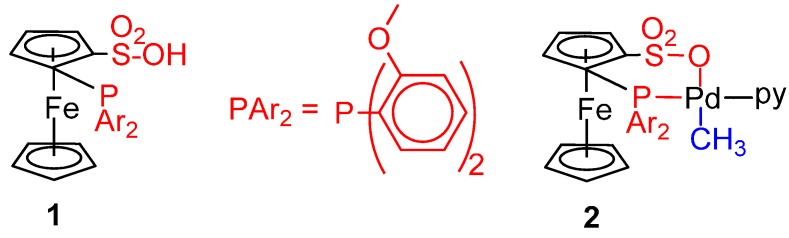
Diarylphosphinoferrocenesulfonic acid **1** for the synthesis of the catalyst precursor **2**.

Ligand **1** in Figure 14 in combination with Pd(AcO)_2_ (1:1) and the preformed precursor **2** in combination with B(C_6_F_5_)_3_ (Pd:B = 1:1.1) were tested for the nonalternating CO-ethene copolymerization. At 110 °C, CO:ethene = 5:60), the ethene extra insertion was relatively high (25%–27%), however the activity is rather low (21–32 g PK(mmol·Pd·h)^−1^ [153].

## 10. Conclusions

The carbonylation of ethene catalysed by Pd(II)-phosphine complexes yields several products, ranging from high molecular weight PKs to MP. The activity and selectivity depend on the bite angle, skeletal rigidity, electronic and steric bulk properties of the ligand. Dppp type ligands are more suitable for the production of perfectly alternating PKs. Substituents at the phenyl rings or at the C_3_ chain may improve the productivity to higher molecular weight PKs. Cationic complexes are active in MeOH or in suspension, whereas the corresponding neutral complexes with coordinating anions, inactive in this solvent, turn to be exceptionally active in H_2_O-organic acid as a solvent. Water soluble catalysts with ligands d-*o*-app-s or d-*p*-app-s are also very active. Substitution of the phenyl groups with bulkier ones, such as *^t^*Bu groups, causes a dramatic change in selectivity as MP formation is largely dominant. Several experimental findings point out that steric hindrance plays a key role in governing the selectivity of the carbonylation of ethene. On one hand, by carefully increasing the steric hindrance of catalysts suitable for the formation of PKs, the productivity increases, due to the destabilization of resting states preceding the insertion of the olefin, thus easing the rate determining step. However, further increase of the steric bulk inhibits more and more the insertion of the olefin, to the extreme case in which after the insertion of just one molecule of each monomer, fast methanolysis of the Pd-acyl intermediate occurs with selective formation of MP as in the case of the dtbpx based-catalyst. Other highly active and selective catalysts for the production of MP have been developed. Catalysts with unsymmetrical ligands having only one P(*^t^*Bu)_2_ group are also very active and selective, which indicates that only one P(*^t^*Bu)_2_ group is required to achieve excellent selectivity and high activity. The CO-ethene copolymerization, catalysed by cationic (P-P) complexes in MeOH, proceeds through both the Pd-H and Pd-OCH_3_ initiators, as unambiguously proven by NMR end group analysis, whereas the monocarbonylation to MP catalysed by monophosphine- or bulky diphosphine-catalysts occurs via a Pd-H initiator only, as proven by synthesis, spectroscopic characterisation of all the key intermediates of the catalytic cycle, as well as isotope labelling experiments and the use of model compounds as precursors. Catalysis with the highly active and selective complexes with the unsymmetrical ligands, having one P(*^t^*Bu)_2_ group involves two geometric isomers for each of the catalytic intermediates in equilibrium as shown in Scheme 6. Since they are highly selective, it is likely that intermediate **1**, with the Pd-acyl moiety *trans* to the P(*^t^*Bu)_2_ group is kinetically dominant over diastereoisomer **2** and dictates the chemoselective product forming step. Neutral complexes with P-O^−^ anionic ligands, which maintain neutrality during catalysis, yield nonalternating PKs, with an excess of ethene in the polymer chain. The origin of the nonalternation has been investigated by experimental studies as well as theoretical ones. The extra insertion of ethene in the P-O^−^ is facilitated because: (i) the formation of Pd-O chelates through coordination of the oxygen atom on the growing chain is less favourable than in the case of cationic P-P catalysts, which yield perfectly alternating PKs; (ii) the ratio of the binding affinities of CO and ethene to the palladium β-chelate is much smaller than in cationic catalysts (50:1 *versus* 10^4^:1); (iii) the lower stability of the **γ**-chelate in the P-O^−^ catalysts facilitates the decarbonylation of its open-chain analogue. Although important results have been achieved, room remains for further development of more efficient catalytic systems.
